# Community Response to Multiple Sound Sources: Integrating Acoustic and Contextual Approaches in the Analysis

**DOI:** 10.3390/ijerph14060663

**Published:** 2017-06-20

**Authors:** Peter Lercher, Bert De Coensel, Luc Dekonink, Dick Botteldooren

**Affiliations:** 1 Medical University Innsbruck, Christoph-Probst-Platz, Innrain 52, Innsbruck A-6020, Austria; 2 Waves Research Group, Department of Information Technology, Ghent University, Technologiepark-Zwijnaarde 15, Ghent B-9052, Belgium; bert.decoensel@ugent.be (B.D.C.); luc.dekoninck@ugent.be (L.D.); dick.botteldooren@ugent.be (D.B.)

**Keywords:** road traffic noise, railway noise, annoyance, mixed sound sources, combined effects, vibration, air pollution, soundscape, environmental health impact assessment

## Abstract

Sufficient data refer to the relevant prevalence of sound exposure by mixed traffic sources in many nations. Furthermore, consideration of the potential effects of combined sound exposure is required in legal procedures such as environmental health impact assessments. Nevertheless, current practice still uses single exposure response functions. It is silently assumed that those standard exposure-response curves accommodate also for mixed exposures—although some evidence from experimental and field studies casts doubt on this practice. The ALPNAP-study population (*N* = 1641) shows sufficient subgroups with combinations of rail-highway, highway-main road and rail-highway-main road sound exposure. In this paper we apply a few suggested approaches of the literature to investigate exposure-response curves and its major determinants in the case of exposure to multiple traffic sources. Highly/moderate annoyance and full scale mean annoyance served as outcome. The results show several limitations of the current approaches. Even facing the inherent methodological limitations (energy equivalent summation of sound, rating of overall annoyance) the consideration of main contextual factors jointly occurring with the sources (such as vibration, air pollution) or coping activities and judgments of the wider area soundscape increases the variance explanation from up to 8% (bivariate), up to 15% (base adjustments) up to 55% (full contextual model). The added predictors vary significantly, depending on the source combination. (e.g., significant vibration effects with main road/railway, not highway). Although no significant interactions were found, the observed additive effects are of public health importance. Especially in the case of a three source exposure situation the overall annoyance is already high at lower levels and the contribution of the acoustic indicators is small compared with the non-acoustic and contextual predictors. Noise mapping needs to go down to levels of 40 dBA,Lden to ensure the protection of quiet areas and prohibit the silent “filling up” of these areas with new sound sources. Eventually, to better predict the annoyance in the exposure range between 40 and 60 dBA and support the protection of quiet areas in city and rural areas in planning sound indicators need to be oriented at the noticeability of sound and consider other traffic related by-products (air quality, vibration, coping strain) in future studies and environmental impact assessments.

## 1. Introduction

Simultaneous or mutual serial exposure to several traffic sound sources is a typical characteristic of modern residential areas worldwide [1]. A recent environmental survey of the German environmental agency [2] revealed that 33% are exposed to more than one source, to two sources (22%) or even to three sound sources (11%). However, in spite of large scale noise mapping across Europe, no data are available at the European level about how much people are exposed to several sound sources. Environmental health impact assessment and planning is still based on single source assessment—likewise the assessment of the economic [3] and health burden [4]. In 2001 Job and Hatfield [5] summarized the status of our scientific knowledge: “Our understanding of the effects of noise from combined sources on reaction, and other potential consequences of noise exposure (e.g., sleep disturbance, cardiovascular disease), is inadequate, despite an array of theories and data pertaining to this issue. Nonetheless, understanding the interactive effects of noise from combined sources is critical to effective regulation”. The current status is not much better. Although a few new proposals have been made [6,7] and summarized [8] to accommodate for mixed source exposure, hitherto, no commonly accepted method can reliably assess the cumulative or interactive effects of multiple noise sources. Among the major models, the “energy summation model” does not take into account the different acoustic characteristics of transportation noises, the “dominant source model” does not fit when sound sources are equally important [1]. Moreover, the judgement of overall or total annoyance—when exposed to multiple sound sources—contains inherent difficulties. The current concept of annoyance [9] and its standardized measurement procedure [10] is focused on single sources. The reporting of total annoyance from several sources requires a perceptual-cognitive integration [11]. This integration constitutes a major challenge and is a recognized cognitive problem for study participants [12]. Consistently, participants judge the total annoyance to be lower when compared with the ratings of the single sources—commonly known as the combined source “paradox” [7,12,13,14]. Moreover, the annoyance data from mixed source exposure stems mainly from two source exposure environments [15,16,17,18,19,20] and few investigated three sound sources in field studies [21]. There are a few more experimental studies [14,22,23,24,25,26,27,28,29], however, the transfer to the community experience is difficult. Recently, a combined experimental and field study [29] evaluated the European Union exposure-response relationships for their ability to predict annoyance from multiple sound sources (road, rail, air) based on the annoyance equivalents model of Miedema [6]. It concluded that this procedure did not well predict annoyance due to combined transportation noises. This is not surprising in the light of the reported large variation of percent highly annoyed for single sound sources such as road, air and railway traffic [30,31]. Fidell et al. [32] coined the term “community tolerance level” (CTL) for the observed large between community differences in annoyance prevalence rates and concluded this to be the result of a combination of acoustic and non-acoustic factors. While the introduction of the CTL improves the prediction in theory—the quantification of the determinants which are responsible for the large differences in “community tolerance” remain to be uncovered in studies with multiple source exposure. Although the potential influence and quantification of non-acoustic factors has been evaluated earlier [33,34]—is knowledge was derived from single sound source studies. Without quantitative knowledge of the underlying reasons for the large deviation in CTL any local and regional planning and implementation of noise action plans is hampered. In a related article, Schomer et al. [30] pointed out that both non-noise variables and non-A-weighted acoustic variables are required to adequately describe the community response. Among the former, personal, attitudinal, situational (e.g., bedroom position, house type), and environmental variables (e.g., air pollution, vibration, green space) can contribute much to the reported annoyance response [35,36,37,38,39]. Among the non-A-weighted acoustic variables strong low frequency, tonal or impulsive components and modulations have been mentioned in the literature [40,41,42]. But also temporal features related to perception (fluctuation and/or emergence) in the context of mixed sound exposure may play a role [43,44,45]. Furthermore, underestimations in noise mapping [46] or a low background sound level [47] are purported reasons. Following this reasoning the CTL may therefore contain also the neglected effects of combined traffic exposure, the general ambient sound quality of the residential area as well as possible experienced simultaneous vibration and air pollution exposure due to these sources. Furthermore, positive aspects of the residential soundscape (quiet sides, greenery) may also counteract negative responses [48,49,50,51,52]. 

In this paper we apply a multimethod evaluation of the mutual influence of mixed traffic sound exposure (highway, main road, railway), and major external (vibration, air pollution, community sound quality, housing, distance, region), internal (noise/air sensitivity, education level), and behavioral-emotional (coping activities, anger) non-acoustical factors on the annoyance response in Alpine communities. The annoyance response in alpine areas was already high in the early nineties [51], has recently shown to exhibit a low CTL [1] and continued to exceed the response of standard curves also after the year 2000 [52].

## 2. Methods

### 2.1. Area, Study Design and Sampling 

The area of investigation covers a stretch of about 40 km in the Lower Inn valley (east of Innsbruck, Austria) and is the most important North-South-access route for heavy goods traffic over the Brenner Pass. It consists of densely populated small towns and villages with a mix of industrial, small business, touristic and agricultural activities. The cross-sectional study was conducted during fall 2005. The primary noise sources are highway and rail traffic. Main roads are an additional important source. These roads link the villages, provide access to the highway and carry also a substantial truck load (up to 15%).

People were contacted by phone based on a stratified, random sampling strategy. The address base was stratified by use of the Geographic Information System (GIS), based on fixed distances to the major traffic sources (rail, highway, main road), leaving a common “background area” outside major traffic activities and an area with exposure to more than one traffic source “mixed traffic.” From these five areas households were randomly selected and replaced in case of non-participation. Selection criteria for people were age between 25 and 75 years, sufficient hearing and language proficiency. An exclusion criterion was duration of living less than one year at this address. Eventually, 1643 persons (35.3% of the original address sample on an individual basis), completed the study. 44.4% did not want to participate or had to be excluded due to other reasons (too young, too old, too short time in the area, insufficient language proficiency, or hearing problems). The rest of the addresses were not valid (commercial etc.), did not have telephone or could not be reached by 3 attempts at different times of the day (e.g., secondary residences, ill or abroad at that time). The participation at the household level was higher (around 50%). The participation proportion was not completely equal across the whole sampling frame: persons living close to traffic lines participated at a slightly higher rate than people living at remote sites. Persons with mixed traffic exposure did participate at a significantly higher rate. Women were more willing to participate (61%). This was the only deviation from a representative population perspective. Informed consent (with signature) was obtained by mail. Only a few refused and their files were instantly deleted. A digital registration, but no Ethical Committee approval was required for this phone survey.

### 2.2. Sound Exposure Assessment

It is well known that the topographic features (“amphitheatre” effect) and the meteorological phenomena (e.g., Foehn winds, inversions) are challenges which require more efforts in exposure assessment [53,54,55]. Three groups of traffic noise sources are covered: highway traffic, traffic on main roads, and railway traffic. A summary of the single and multiple sound source level distribution is provided in the Supplementary Material (Figure S1a,b). For highway traffic the yearly average load (light and heavy vehicles) is combined with an average diurnal traffic pattern. Traffic frequency data on main roads were supported by actual counting data. Road traffic noise emission is calculated on the basis of the Harmonoise source model [56]. Railway noise emission is extracted from a typical day of noise immission measurements at close distance to the source.

Sound modelling was applied with an adapted version of ISO9613 (Bass3) to account for the topographic and meteorological features. The model includes up to four reflections and two sideway diffractions [57,58].

In parallel an extensive noise monitoring campaign was conducted to check the validity of these simulations. At 38 locations sound levels were recorded for over one week during winter (October to January) and during summer (June to August). In addition, the predicted sound pressure levels resulting from PE-modelling have been evaluated against these long-term measurements [58,59]. 

Classical indicators of day, evening, night exposure and Lden were calculated for each source and total sound exposure (energy summation) at several points on the facade of the building of the survey participants. Moreover, indicators of fluctuation and emergence were derived for all sources to evaluate additional acoustical effects on perception [43]. Fluctuation is defined as the difference between the source event (L1 for highway, L5 for main roads, L10 for railway) and the source background level (L90 for highway, L99 for main roads, L90 for railway). Emergence is defined as the difference between the source event (L10 for highway, L5 for main roads, L10 for railway) and the overall background level originating from all natural and traffic sources except the source under study (L90 for highway, L99 for main roads, L90 for railway). In most of the present analyses Lden from the most exposed façade is applied. In some analyses, where methodological constraints (multicollinearity) did not prohibit its application, emergence is studied as alternative acoustical exposure indicator.

### 2.3. Air Pollution Exposure Assessment

Research from Austria, Switzerland and from the ALPNAP-study group have shown that due to the specific meteorological, climatic and topographic conditions in alpine valleys, the same amount of emission generates up to four times higher air concentrations at the receiver point than in flat land [60,61,62,63,64]. The air pollution assignments were prepared by the Institute for Internal Combustion Engines and Thermodynamics, Technical University Graz, Austria. Annual means for NO_x_, NO_2_ and PM10 were calculated for an area 27 km (W–E) × 23 km (N–S) east of Innsbruck. For the air quality assessment about 300 flow fields were calculated with the Graz Mesoscale Model (GRAMM) meteorological model [64,65]. For each flow field a dispersion calculation with the Lagrangian particle model GRAL [66,67] was carried out on a very fine horizontal resolution of 10 × 10 m² and vertical on 2 m resolution. The fine resolution enables to resolve the dispersion close to strong sources such as highways. The model system uses special algorithms to account for low wind or calm conditions [68,69]. Each run was weighted due to its meteorological classification and frequency. Traffic emissions were modeled using the network emission model NEMO [70]. Thereafter, annual, summer and winter means were calculated by post processing and weighting the numerous dispersion calculations. The NO_x_ to NO_2_ conversion is calculated according to the scheme of Romberg et al. [71]. 

Because the model calculates the exposure resulting from specified emissions such as traffic, domestic heating etc. a residuum results when comparing simulations versus observations. This residuum or so-called background value which is the abscissa of the regression analysis is attributable to not accounted emissions or secondary aerosol formation or regional transport not accounted in these micro-scale dispersion calculations. The simulation results were compared with 7 air quality stations located in the Inn Valley. The background values within this study were height corrected according to Seinfeld and Pandis [72]. Calculated annual NO_2_ values (corrected for the results of the measurements) for each of the participant's home were assigned by GIS and used in this analysis.

### 2.4. Questionnaire Information

The questionnaire covered socio-demographic data, housing, satisfaction with the environment, general noise annoyance, attitudes toward transportation, interference with activities, coping with noise, occupational exposures, lifestyle, reported sensitivities, health status, selected illnesses and medications. The phone interview took about 15–20 min to complete. Education was measured with five grades (basic, skilled labor, vocational school, A-level, University degree). The last two grades were combined in the category “higher education. Health status was judged on a standard 5-grade scale. In the analysis we used very good (1), good (2) and less than good (3 + 4 + 5). Noise sensitivity, sensitivity to vibration and air pollution was asked with a 5-point Likert-type question. High was defined by the two upper points on the scale (4 & 5) in the analysis. Noise annoyance was measured with a 5-point verbal scale according to ICBEN [10] and ISO standards (ISO TC 43/SC 1 2002-02). Annoyance was specifically asked for highway, main road and railway. One question was on overall/total annoyance by all traffic sources (roads and rail). This question was asked *before* the rating of the specific sources. In the present analyses, highly annoyed (HA) was defined by responses to the two upper points (4 + 5) of the 5-point verbal scale, (at least) moderately annoyed (MA) included the three upper points (3 + 4 + 5). Further annoyance questions were related to vibration (due to railway and road traffic) and to air pollution (traffic exhaust and dust/soot). Any reported vibration annoyance on the scale (2 + 3 + 4 + 5) was counted in the analysis while high annoyance due to air pollution was defined by the two upper points on the scale (4 & 5). 

Active and emotional coping efforts were assessed by a sum score based on 13 items (used in Botteldooren and Lercher [73]). Anger and helplessness reactions towards the experienced traffic at home were inquired by a 5-point frequency scale (never = 1, rarely = 2, sometimes = 3, most of the time = 4, always = 5). These responses were summarized for the analysis into 3 categories (never, 2 + 3, 4 + 5). The area characteristic (urban, suburban, and rural) was defined based on residential pattern and community size by a geographer. To assess the environmental quality of the larger living area we used the German question 5 of the Eurobarometer 51.1: “Where you live, do you have very much reason, quite a lot of reason, not very much reason or no reason at all to complain about... ?” related to noise and air pollution. In the analysis we lumped the 2 lower categories. In a few full models a sum score of sleep disturbance was entered (Cronbach’s alpha = 0.82). It consists of 5 frequency items (never, rarely, several times/month, several times/week, nearly daily) related to sleep problems (sleep onset, awakening, fall asleep again, early awakening, tiredness in the morning). Life satisfaction was measured and scored according to the world life satisfaction survey [74].

### 2.5. Statistical Analysis

Exposure and survey data were linked through the Geographical Information System and statistical analysis was conducted with R-Software [75]. Dichotomous variables for Table 1 were examined by the Pearson Chi-square test. For numeric type data medians and inter-quartile ranges are presented and the *p*-values of the Wilcoxon Rank Sum test are reported.

Reliability analyses (Cronbach’s alpha) for the scales used were conducted with the R-package psych [76]. Depending on the annoyance outcome (dichotomous (HA + MA) or full annoyance scale), exposure-effect relationships were modelled with multiple logistic (lrm-module) or linear (ols-module) regression techniques using Harrell’s RMS-library [77]. To account for potential non-linearity in the sound indicators splines were applied where appropriate. Approximate 95% confidence intervals were estimated using smoothing spline routines with three knots and the exposure-effect plots were generated with the RMS-library. Predicted probabilities are derived from the estimated odds with a specific function in the RMS-library (plogis). The predicted probabilities in the exposure-effect estimations and plots are adjusted to the median (continuous variables) or the reference category (non-continuous variables) of the other variables in the model. We used several strategies. A basic model (sound source, age, gender, education, health, noise sensitivity) assessed the relation between a single sound source and annoyance with one additional sound source. The additional sound source was entered as continuous variable or as difference measure (using reasonable dBA categories for the respective sources). In a further step all three sound sources entered the model. With an extended model including further potentially confounding/moderating variables (distance to source, annoyance due to vibration, air pollution, air sensitivity, area quality, required coping and emotional reactions, life satisfaction) to evaluate the results of the basic model. Formal tests for interactions between sources were conducted with the basic models. No significant multiplicative interactions (e.g., rail sound*highway sound) were found (*p*-level lowered to 0.2) and not further tested with the full models.

Analyses and model building was guided by prior substantive knowledge, previous experiences and statistical guidance outlined in [78,79]. Model assumptions were checked and sensitivity analyses were carried out after [79]. The full scale annoyance variable was not normally distributed (peak at no annoyance), but the further requirements were well met. Except a slight homogeneous heteroscedasticity was noted, but no model change was observed, when a correction (robcov-procedure of the rms-package) was applied. Specifically, the final models are evaluated against multiple discrimination criteria (Akaike information criterion, Bayesian information criterion, R^2^, model χ^2^, Somers’ Dxy, Spearman’s ρ, Gamma, Tau-a, C (area under “Receiver Operating Characteristic” curve). Based on these discrimination and accuracy criteria the best model was chosen balancing against potential collinearity. Thorough testing for collinearity was required, as highway and railway sound exposure was highly correlated (*r* = 73). The alternative sound indicators (fluctuation and emergence) correlated even higher (up to 0.88). We took as first-hand indicators the calculated VIFs from the rms package and the enhanced indicator from the car package (GVIF^(1/(2 × df)^). In a second step an extended check was made by investigating the various predictor matrices for Eigenvalues and the determinants of the covariance matrix. With the R package perturb [80]—which implements the classic approach of Belsley et al. [81]—condition indices and the variance decomposition proportions were studied to detect the responsible predictor variables. Based on these analyses the total sound level and the fluctuation indicators had to be removed from a few models to guarantee reliable overall model estimations.

## 3. Results

### 3.1. Sociodemographic Sample Characteristics 

Univariate categorical socio-demographic, health, housing, area and reaction characteristics of the full sample are described in relation to the total annoyance response in Table 1. Only gender and house type did not differ significantly with respect to total annoyance. The highest and lowest age groups showed lower annoyance, while two groups in between (35–44, 55–64 yrs) exhibited higher annoyance. The educational differences did not follow a linear pattern of length of school years. Person’s sensitivities, poorer health status, and reactions were significantly related to total annoyance. Also those living closer to the traffic sources were significantly higher annoyed. Among the source, residents annoyed by highway showed the largest high total annoyance percentage.

Among the relation of the continuous variables with the total annoyance reactions duration of living (years at the current home) was not significantly different between the highly and less annoyed and compared with other surveys relatively high (median of 16 yrs, interquartile range (IQR) of 7 and 31 yrs). As expected, people highly annoyed lived closer to the highway and railway. Contrary, the relation with the distance to the main road was not significant. It even indicates—although the median sound levels from the main road are low—the sound is perceived as annoying (by the peaks) due to the lower mean back-ground sound level of (L95 = 34.5 dBA) compared with 41.3 dBA from highway. However, the sound exposure levels of the highway and railway are significantly higher than the one from the main road (see Table 2 and Figure S1). Also the air pollution levels are higher in the highly annoyed group (Table 2). Furthermore, life satisfaction is significantly lower and the sleep disturbance and coping efforts score are significantly higher among the highly annoyed by all sources (Table 2).

### 3.2. Exposure Variables and Other Continuous Model Characteristics

Table 2 gives a statistical description of continuous model variables broken down by total noise annoyance. All acoustical indicators show significant relations (*p* < 0.001) with high annoyance ratings. Distance to highway and railway track is negatively associated with annoyance (*p* < 0.001) while distance to the main road slightly failed significance (*p* = 0.071). Annual air pollution (indicator NO_2_ levels) is significantly associated with high annoyance ratings (*p* < 0.001). Satisfaction with life score is negatively related (*p* < 0.001) and sleep disturbance and coping scores are positively associated with higher annoyance (*p* < 0.001). Longer duration of living in the home is not significantly associated with higher annoyance (*p* = 0.163).

### 3.3. Sound Source Exposure Response (ER) Models: Unadjusted

**Figure 1:**
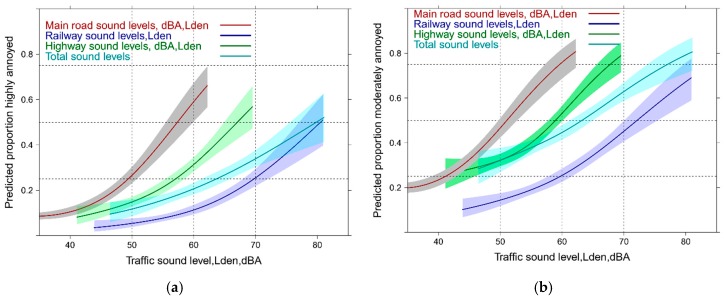
Predicted proportion of high and moderate annoyance associated with single source specific and total sound exposure (total annoyance). (**a**) Proportion highly annoyed; (**b**) Proportion moderately annoyed.

In Figure 1 the exposure response (ER) relations for all single sound sources (source annoyance) and the total sound exposure (total annoyance) are graphically summarized. Independent of the cutoff level for annoyance, main road exposure is more disturbing than highway and railway. Reported total annoyance by total sound exposure falls midways between highway and railway for the highly annoyed cutoff (Figure 1a), while for the moderately annoyed (Figure 1b), the total sound exposure lies closer to the highway experience below 60 dBA,Lden and approaches successively the railway response towards higher sound levels. Figure 1 indicates smaller overall sound exposure levels for main road and highway compared with the railway exposure in this survey. Note: all source model terms are highly significant (*p* ≤ 0.0001) but the pseudo-R² is quite low (main road: 0.15; railway: 0.12; highway: 0.10). Main road and highway exhibit an additional significant non-linear component (main road: *p* < 0.001; highway: *p* = 0.0082). A note of caution regarding the railway response: extensive noise abatement measures were implemented in the years preceding the survey (see also the Discussion section).

**Figure 2:**
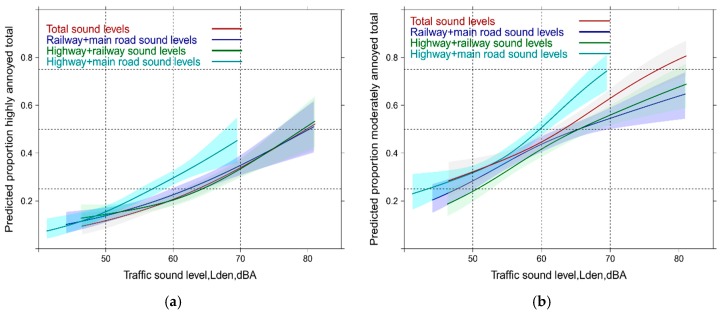
Predicted proportion of total high and moderate annoyance associated with mixed sound level exposure. (**a**) Proportion highly annoyed; (**b**) Proportion moderately annoyed.

Figure 2 reveals the total annoyance experience with the mixed sound source exposure as obtained from logistic models for each of these combinations. Note: the outcome in this graph is necessarily “total annoyance.” Interestingly, the combined main road and highway exposure significantly departs from the other combinations between 55 and 60 dBA, Lden with both annoyance cutoffs. This is related to the specific exposure situation in the study area where high exposure levels for railway noise are encountered and thus increased percentages of highly annoyed are observed at lower exposure levels for highway and main road levels. The total sound exposure annoyance curve is only higher with the lower annoyance cutoff (Figure 2b) compared with other combinations—but is still significantly lower than the combined main road and highway exposure response (ER) which increases from 50% to 75% moderately annoyed between 60 and 70 dBA,Lden. Note: the ANOVA tables and the R² of typical regression models are provided (Tables S1–S8) in the Supplementary Material.

Likewise—although the mixed source model parameters are highly significant (*p* ≤ 0.0001)—the pseudo-R² is lower than for the single sound source models for specific annoyance (main road + railway: 0.06; highway + railway: 0.06; highway + main road: 0.06; total sound exposure: 0.07). Only the highway + railway combination exhibits a weakly significant non-linear component (*p* = 0.0407). The model for total annoyance with the Lden for railway, highway and main road entered separately also outperforms the combined noise models (R^2^ = 0.085).

**Figure 3:**
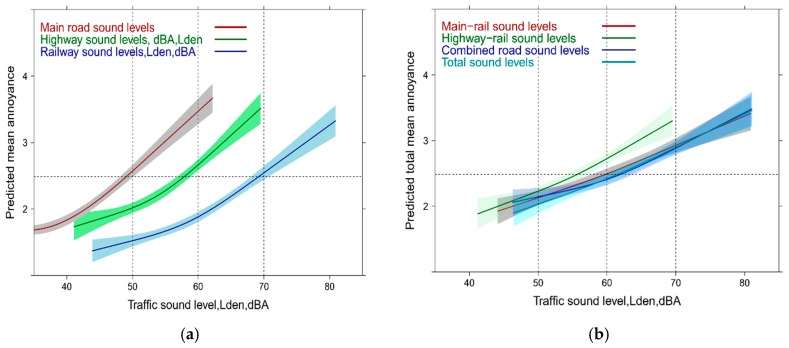
(**a**) Predicted mean annoyance by specific sound sources associated with single sound source Lden; (**b**) Predicted total mean annoyance by multiple source exposure Horizontal line = mean total annoyance.

Sometimes, critique is issued about possible restrictions by judging the public health impact only with highly annoyed. This could be particularly critically in the case of multiple exposure where people have known difficulties to judge overall annoyance. We therefore decided to evaluate also the relationships with the whole span of the ICBEN 5-point scale. We can observe large differences (Figure 3a,b) between the sources in the single exposure situation (similar as in Figure 1 with the HA and MA outcome). In the multi-exposure context the model using the combined highway-main road Lden departs again from the models with other source combinations between 55 and 70 dBA. Notably, the slope becomes shallower and a higher mean annoyance response is seen at lower levels—which is not so evident from the HA-approach (Figure 2a).

Since we have explored alternative/supportive sound indicators (fluctuation, emergence) in an earlier proceeding article [49] we analyzed the relation of fluctuation and emergence for all sources only on the total annoyance response. However, due to its high correlation with the Lden of all sources the models were too unstable (multicollinearity). Therefore, we ran the models with the alternative indices only. Even then the correlation among all alternative indices of the 3 sources was too high (*r* = 0.8–0.88). Only the models with the emergence indicators were stable enough. The adjusted results are reported in the next section due to negligible differences between the unadjusted and the adjusted models.

### 3.4. Sound Source Exposure Response Models: with Demographic/Health and Source Adjustments

#### 3.4.1. Sound Source Exposure Response Models: with Total Annoyance (All Sources)

**Figure 4:**
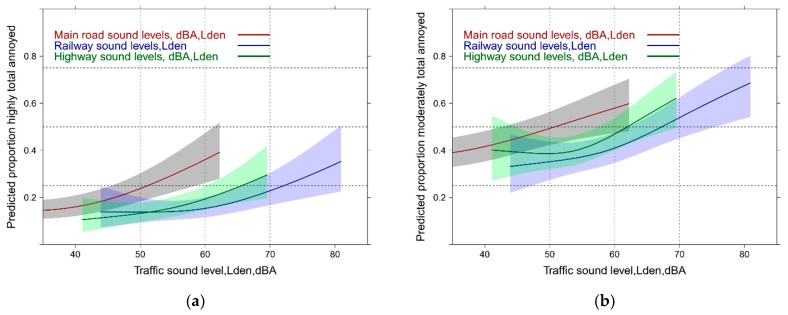
Predicted proportion of total annoyance associated with sound source level (Lden, dBA). (**a**) Proportion highly annoyed; (**b**) Proportion moderately annoyed. Adjusted for age, sex, education, noise sensitivity, health status and the other sound sources.

These models differ essentially from the models in Section 3.3. Firstly, the model adjustments for age, sex, education, noise sensitivity, and health status. Secondly, all sound source indicators were included in the model (see Supplement: Tables S1–S8 for details of the regression model). Consequently, the total annoyance judgement was used as outcome. With this analysis approach we expect to get more insight into the “combined noise source paradox.” Compared with Figure 1 the individual slopes of total annoyance versus individual source Lden become shallower (Figure 4a,b) and approach each other more closely –similarly to the results of the mean total annoyance versus overall Lden in the mixed source analysis (Figure 3b). Note that this is not due to the adjustments but due to the fact that total annoyance is modelled. However, even after adjustment for highway and railway sound the total noise annoyance is higher at low levels of main road Lden, notably through its departure between 50 and 60 dBA. At both sound level points, annoyance by the main road is significantly higher compared with highway (*p*-at50 ≤ 0.0001; *p*-at60 = 0.0004) and railway exposure (*p*-at50 = 0.0014; *p*-at60 ≤ 0.0001). Highway and railway sound levels induce about the same annoyance below 55 dBA. At 60 dBA highway is significantly more annoying (*p* = 0.0045) but no longer around 70 dBA (*p* = 0.3087). Nearly identical shapes of the exposure response curves are obtained in the model with the moderately annoyed outcome (Figure 4b). Notably, a more than doubling of the annoyance response is observed and substantial proportions are already moderately annoyed at levels between 45 and 50 Lden, dBA, as the other sources determine total annoyance in that case.

**Figure 5:**
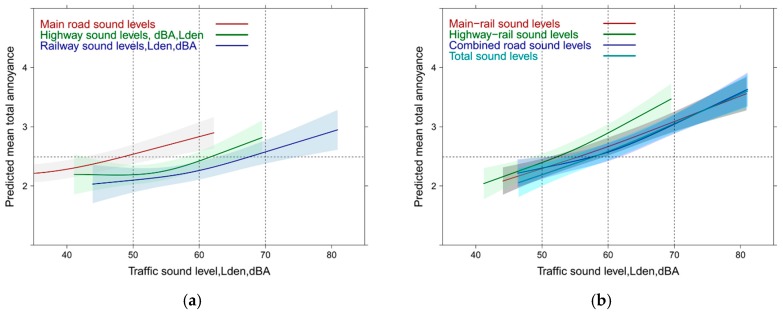
(**a**) Predicted mean total annoyance associated with single sound sources; (**b**) with multiple source exposure. Adjusted for age, sex, education, noise sensitivity, health status and the other sound sources. Horizontal line = mean total annoyance.

With the mean annoyance outcome approach the single (adjusted) source results are similar compared with the HA + MA-outcome in Figure 5—regarding both—the annoyance of the sources and also the slope of the curve. In the case of the mixed exposure the total exposure level curve is not different from the other relationships—again, except for the highway-rail combination (compare with Figure 3b). This is contrary to what we have observed in the HA + MA-outcome analysis, where the highway-main road combination yields higher annoyance around 60 dBA than all other source combinations including the total sound exposure indicator.

#### 3.4.2. Sound Source Exposure Response Models: with Total High and Moderate Annoyance (Mixed Sources)

**Figure 6:**
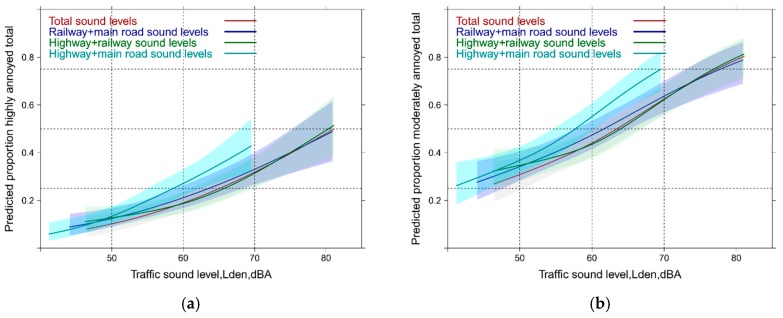
Predicted proportion of total annoyance associated with sound from mixed sources. (**a**) HA = Proportion highly annoyed; (**b**) MA = Proportion moderately annoyed. Adjusted for age, sex, education, noise sensitivity, health status and the other mixed sources.

In Figure 6a,b the equivalent results are presented for the adjusted mixed sources models. The results mimic the exposure response curves of the unadjusted model shown in Figure 2. The road combination reveals again the steepest slope and reaches 25% highly annoyed around 60 dBA, Lden (Figure 6a). The other source combinations (including the total exposure) do not show distinguishable response curves.

The adjusted mixed source model parameters were all highly significant and the model pseudo R² approximately doubled for all mixtures (R² = 0.12–0.13). There is no significant non-linear component.

#### 3.4.3. Sound Source Exposure Response Models: with Total High and Moderate Annoyance Related to Emergence Indicators

**Figure 7:**
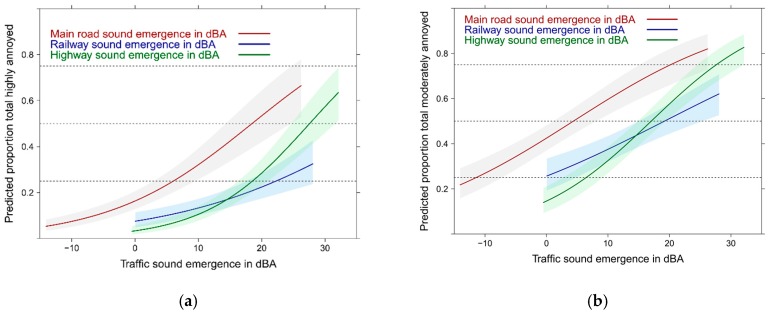
Predicted proportion of total annoyance associated with the emergence level of the sound sources (dBA). (**a**) Proportion highly annoyed; (**b**) Proportion moderately annoyed. Adjusted for age, sex, education, noise sensitivity, health status, and the other sound sources.

Further model testing were conducted by using all emergence indicators. With the emergence indicators –instead of the Lden–for the 3 sources in the logistic regression model the total annoyance follows a similar pattern (Figure 7). 

The proportion of highly and moderately annoyed by main road stands out. Notably, the proportion highly annoyed increases more rapidly with the emergence indicator for highway and main road noise than with the emergence indicator for railway noise.

#### 3.4.4. Sound Source Exposure Response Models: with Mutual Exposure of Another Source (Single Source High Annoyance)

**Figure 8:**
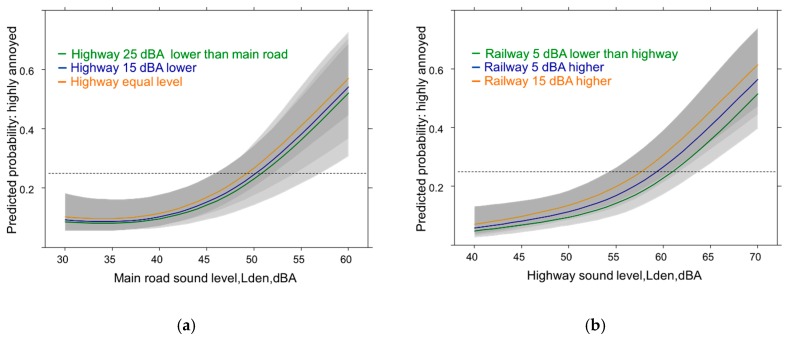
Predicted proportion of high source annoyance associated with level differences to the other source (in dBA). (**a**) Main road & highway; (**b**) Highway & railway. Adjusted for age, sex, education, noise sensitivity, health status and the other sound source difference.

Eventually, adjusted sound source difference models were evaluated for additional insight into the mutual dependence of source annoyance on the other source level experienced in a sound combination. The results suggest a small (but non-significant) annoyance effect of higher levels of the other source (Figure 8a,b) for these exposure combinations while lower sound levels of the other source are related to lower annoyance (Figure 8b: *p* = 0.06).

**Figure 9:**
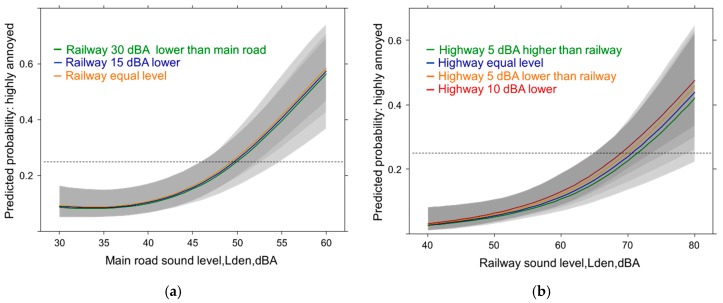
Predicted proportion of high source annoyance associated with level differences to the other source (in dBA). (**a**) Main road & railway; (**b**) Railway & highway. Adjusted for age, sex, education, noise sensitivity, health status and the other sound source difference.

In spite of large differences in level no real annoyance effect is observed when main road (Figure 9a) is experienced together with railway exposure. On the other hand annoyance from railway exposure (Figure 9b) tends to be affected by highway sound levels: When the highway shows equal or higher sound levels then annoyance due to railway is slightly lower (n.s.).

**Figure 10:**
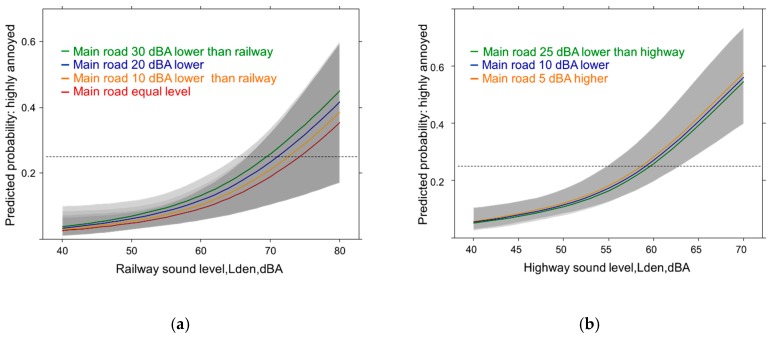
Predicted proportion of high source annoyance associated with level differences to the other source (in dBA). (**a**) Railway & main road; (**b**) Highway & main road. Adjusted for age, sex, education, noise sensitivity, health status and the other sound source difference.

In Figure 10 the effect of the sound levels of the main road on highway and railway annoyance is shown across the sound levels of these two sources. When the sound levels of the main road are much lower than the railway exposure (Figure 10a) then the annoyance response due to railway sounds is higher and increases with the level experienced from the rail exposure. Due to the large variation also the extreme difference is statistically not significant (*p* = 0.15). In the case of the highway exposure, however, the main road sound level has no effect at all on the highway annoyance response (Figure 10b).

#### 3.4.5. Sound Source Exposure Response Models: with Total High and Moderate Annoyance at Night (All Sources)

**Figure 11:**
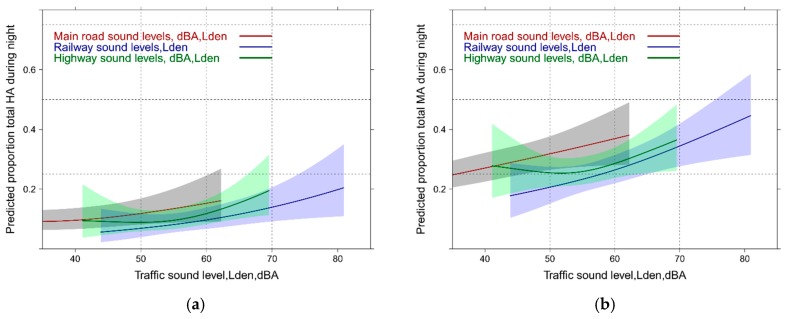
Predicted proportion of nighttime total annoyance associated with sound source level. (**a**) HA = Proportion highly annoyed; (**b**) MA = Proportion moderately annoyed. Adjusted for age, sex, education, noise sensitivity, health status and the other sound sources.

In Figure 11 annoyance by nighttime is analyzed in the adjusted model when all sound sources are considered together. We observe: the proportion of nighttime annoyance due to all sources is lower compared with the overall annoyance in Figure 1. In addition, the annoyance determination by the individual sound sources is less distinct. The difference between high annoyance by main road to highway and railway is smaller at 50 dBA (*p* = 0.0688; *p* = 0.0318) and no more significant at 60 dBA ((*p* = 0.3265; *p* = 0.0741). Neither at 60 nor at 70 dBA a significant difference can be observed between the annoyance responses due to highway and railway sound exposure. 

Only with the larger group of moderately annoyed the source difference is still significant for a few sound levels: main road vs. highway/railway at 50 dBA (*p* = 0.0137; *p* = 0.0018) but smaller at 60 dBA (*p* = 0.0896; *p* = 0.0222). No significant differences are obtained at 50, 60 or 70 dBA between highway and railway. Again, like in Figure 3a more than doubling of the annoyance proportion is seen when the cutoff includes also the moderately annoyed persons.

### 3.5. Sound Source Exposure Response Models Including Contextual Variables

The sound models with full accommodation of contextually important variables exhibited a much higher variance explanation reflected through larger model pseudo R² values (see Supplementary Material Tables S1–S8 for R² of the models). This means the pseudo R² grew from an observed range of 0.12–0.15 in the basic adjusted models up to 0.55 in the full contextually enriched sound source models.

#### 3.5.1. Highway Sound Exposure: Full Contextual Models with High Annoyance

**Figure 12:**
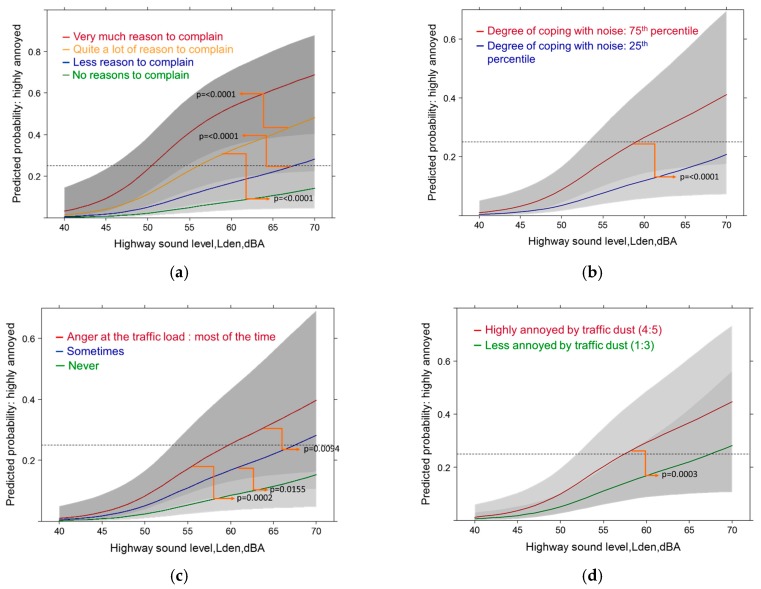
Predicted probability of high annoyance associated with highway sound exposure by: (**a**) complaints about community soundscape; (**b**) coping efforts. Models are adjusted for age, gender, education, health status, sleep score, distance to sources, sensitivity to noise/air pollution, living duration, anger at traffic load, coping (not **b**) perceived vibration from road, general satisfaction with life, region and air pollution (annual NO_2_ values); (**c**) anger about the traffic load; (**d**) perceived annoyance by dust/soot. Models are adjusted for age, gender, education, health status, sleep score, distance to all sources, sensitivity to noise and air pollution, living duration, complaints about community soundscape, coping, perceived vibration from road, general satisfaction with life, region and annual NO_2_ values.

With full accommodation of contextually important variables the model pseudo R-squared value increase to 0.55 in the highway model. By far the largest statistical contribution (measured by Wald statistic) was made by complaints about the community soundscape and the required amount of coping actions (*p* ≤ 0.0001). In Figure 12a,b the significant differences of the variable values are displayed. The second largest group contribution was made by the highway sound level itself (*p* = 0.0003), anger at total traffic load (*p* = 0.0007, Figure 12c) and through the perception of dust/soot at home (*p* = 0.0003, Figure 12d). Also distance related variables (distance from highway (*p* = 0.0074) and other traffic tracks (*p* = 0.0074) made highly significant contributions. Overall, the importance of the socio-demographic and health related variables decrease in all full models. However, overall satisfaction with life (*p* = 0.0138), gender (*p* = 0.0200) and age (*p* = 0.0376) where still significant in the highway model. Among the health variables only the sleep quality score showed borderline significance (*p* = 0.0594). Sensitivity to noise or air pollution was no longer significant in the full models—indicating—that by including further contextual variables related to adaptation activities the individual sensitivity is already accounted for (assuming, the sensitives are active copers).

#### 3.5.2. Railway Sound Exposure: Full Contextual Models with High Annoyance

**Figure 13:**
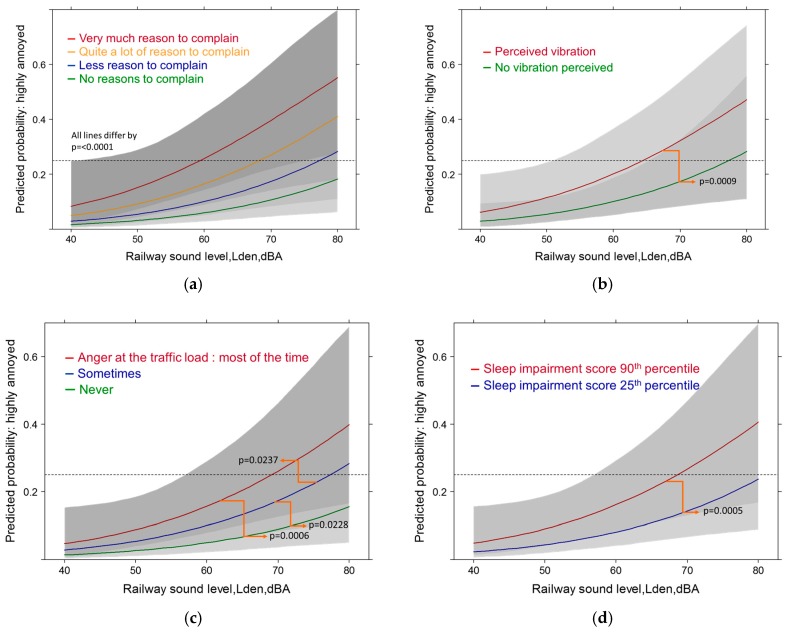
Predicted probability of high annoyance associated with railway sound exposure by (**a**) complaints about community soundscape; (**b**) perceived vibration. Models are adjusted for age, gender, education, health status, sleep score, distance to all sources, sensitivity to noise and air pollution, living duration, anger at traffic load, coping activities, perceived air pollution from road, general satisfaction with life, region. Predicted probability of high annoyance associated with railway sound exposure by (**c**) anger about traffic load and (**d**) perceived vibration. Models are adjusted for age, gender, education, health status, sleep score, distance to sources, sensitivity to noise/air pollution, living duration, complaints about community soundscape, anger at traffic load (not **c**), perceived vibration (not **d**), perceived traffic air pollution, general satisfaction with life, region).

The full railway model shows the same picture overall, however, in a few details there are relevant differences to the highway model. The model pseudo R-squared value is significantly smaller (0.37). The railway sound level is the second largest contributor (*p* = 0.0002). The sleep quality score (*p* = 0.0005) follows shortly before perceived vibration from rail traffic (*p* = 0.0009) and anger at the traffic load (*p* = 0.0022). Among the distance related variables only the vicinity to the various traffic tracks is significant (*p* = 0.0022). Notably, for railway noise the coping efforts were no longer a significant variable. Not surprisingly, air pollution variables were not important contributors—but perceived vibration was. Contrary to the highway model, the statistical importance of socio-demographic and other health related variables decreased and were no longer significant in the model. The exposure relationship of the railway sound level with high annoyance displays the importance of the degree people complain about the overall community sound climate (Figure 13a). In Figure 13b the relationship illustrates that additional perception of accompanying vibration from rail pass-bys contributes significantly to an increase in the predicted prevalence of highly annoyed persons. Figure 13c points to the significant importance of emotional feelings (anger) towards the overall experienced traffic load. Figure 13d illustrates the additional modifying effect of impairment of sleep quality on the relationship of railway noise with high annoyance.

As the sleep quality was independently rated in the health section of the questionnaire and not asked within the context of experienced noise exposure—this underlines the contribution of (often behaviorally unnoticed) sleep disturbance to the generation of annoyance expressions.

#### 3.5.3. Main Road Models: Full Contextual Models with High Annoyance

**Figure 14:**
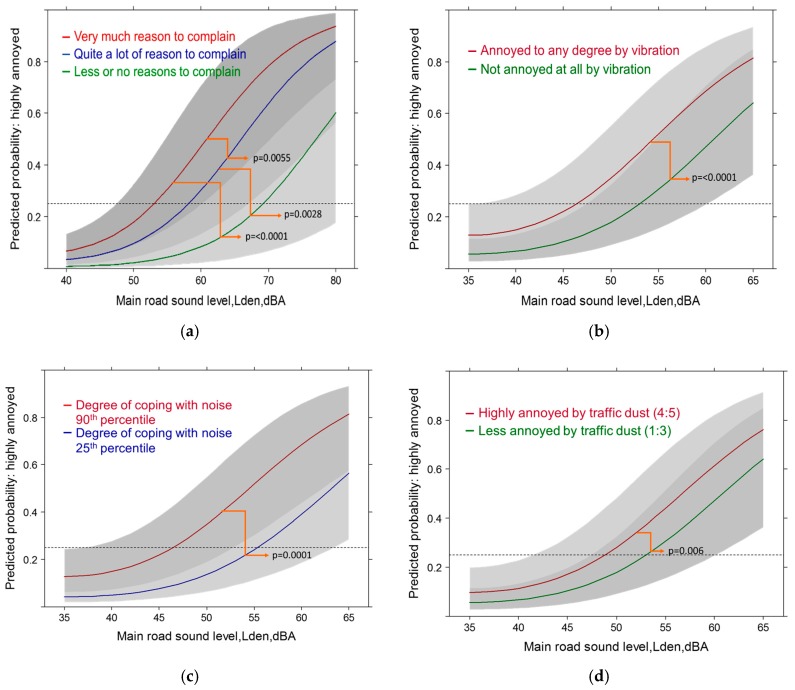
Predicted probability of high annoyance associated with main road sound exposure by (**a**) complaints about community soundscape and; (**b**) perceived vibration. Models are adjusted for age, gender, education, health status, sleep score, distance to sources, sensitivity to noise and air pollution, complaints (not **a**), living duration, anger at traffic load, perceived vibration (not **b**), perceived air pollution from road, general satisfaction with life, location of traffic tracks, region and air pollution (annual NO_2_ values). Predicted probability of high annoyance during the past year associated with main road sound exposure by; (**c**) coping efforts and (**d**) perceived dust/soot exposure. Models are adjusted for age, gender, education, health status, sleep score, distance to all sources, sensitivity to noise and air pollution, coping score (not **c**), living duration, anger at traffic load, perceived air pollution from road (not **d**), general satisfaction with life, location of traffic tracks, region and air pollution (annual NO_2_ values).

The results of the main road model resemble more closely the highway model. In this model, however, the sound level is by far the strongest determinant of the annoyance response. The next best performers are the dissatisfaction with the community soundscape (*p* ≥ 0.0001) and the perception of vibrations (*p* ≥ 0.0001) from the pass-bys of traffic (Figure 14a,b). 

Required coping activities (*p* = 0.0001) and disturbance by dust/soot (*p* = 0.0060) follow (Figure 14c,d). Similar to the other source models, the location of other traffic tracks is another significant factor (*p* = 0.0138). Other source distance variables were not significant contributors. Interestingly, anger about the traffic load was not important with the main road. Also other health related and socio-demographic factors were not associated with high annoyance due to noise from main roads.

### 3.6. Linear Full Regression Models with Continuous Annoyance Ratings: Mixed Sources

**Figure 15:**
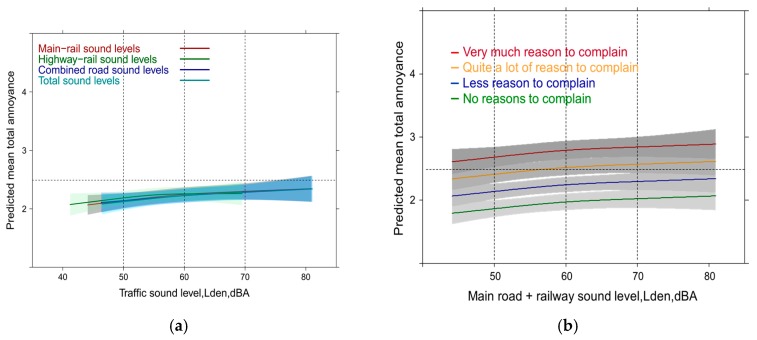
(**a**) Predicted mean total annoyance associated with multiple source combinations; (**b**) Predicted mean total annoyance associated with the largest single predictor (dissatisfaction with community soundscape) main road-rail combination. Adjusted for age, sex, education, noise sensitivity, health status, the other sound sources, anger at traffic load, perceived vibration and perceived dust/soot from traffic, complaints about air pollution at the community level and complaints about community soundscape (not **b**). Horizontal line = mean total annoyance.

When all contextual factors are accounted for in the multiple source combination situation, only a small contribution is being made by exposure levels to the mean annoyance outcome. The slope of the curve is therefore very shallow. Only the railway-main road combination (*p* = 0.01) and the total sound exposure (*p* = 0.03) are still significant in the models. The overall R² of the model is high and very similar in all models including the combination of two sources or the combination of three sources as an exposure indicator (0.58). The main contributing factors are similar to the full models for source specific high annoyance in the previous section: dissatisfaction with the community soundscape and coping efforts are by far the most important in terms of the Wald statistic (chi-square statistic). It follows anger towards the traffic load, perception of dust/soot and vibration. All contributors are highly significant (*p* ≥ 0.0001). Figure 15a shows the shape of the average relation of all mixed source combinations with the mean annoyance response. The curves are not anymore distinguishable. Instead the main contributing factors explain much more variance at any sound level. Figure 15b displays the relation of the railway-main road exposure combination broken down by the largest contributor of the model: dissatisfaction with noise at the community level. Interestingly the model did not show any significant interaction effect between exposure and reason to complain about the noise at the community level.

**Figure 16:**
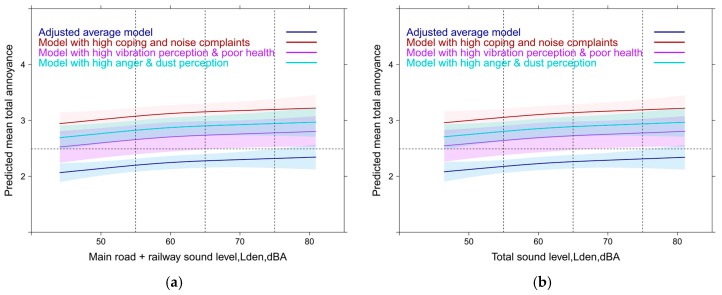
Predicted mean total annoyance associated with main road-railway source combinations (**a**) Horizontal line = mean total annoyance. Adjusted for age, sex, education, noise sensitivity, health status, complaints about air pollution at the community level + the respective other factors in the specific model; (**b**) Predicted mean total annoyance associated with the total sound level combination (same adjustments).

If you consider more combinations of relevant variables then the predicted mean response can be higher or lower than the average relationship. Figure 16a,b show a few predictor combinations for the main road-railway combination and the total sound level situation. The absence of significant interaction effects shows that Lden has no effect on coping or noise complaints (see discussion about the limitation of causal interpretations). The differences between the sound combinations are negligible as already observed in the overlaid summary curve of all sound exposure combinations above (Figure 15a).

### 3.7. Total Exposure Response Models Including Contextual Variables: High and Moderate Annoyance

All models have the overall response to the total sound source exposure in focus. All models include the same variable set, which was checked for multicollinearity. Note, for this reason, not all predictors could be included in these models, compared with the full models that contained only one exposure source (Section 3.5). Some predictors did not contribute anymore and/or were suspects for collinearity (like noise/air pollution sensitivity, air pollution predictor, life satisfaction, sleep score, region, and distance to other traffic tracks). The variance explanation (pseudo R²) of the models remains of similar size.

#### 3.7.1. All Source Total Exposure Response Models Including Contextual Variables

**Figure 17:**
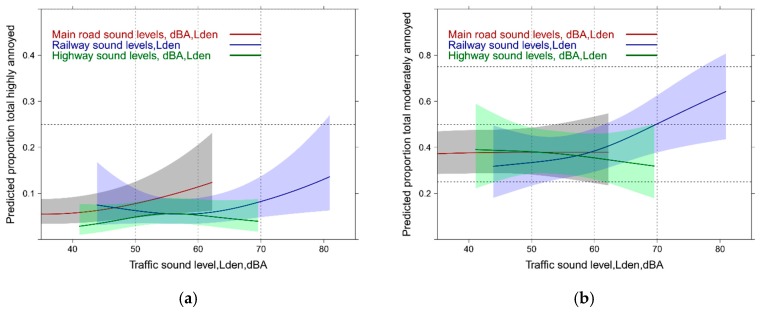
Predicted proportion of total annoyance associated with sound from all sources. (**a**) Proportion highly annoyed; (**b**) Proportion moderately annoyed. Models are adjusted for age, gender, education, health status, complaints about community soundscape and air pollution, perceived vibration, perceived dust/soot from road, coping, anger at traffic load and the respective other sound sources.

Compared with the basic adjusted models the dependence of total annoyance on Lden of individual sources becomes weaker for both, the high and moderate annoyance response (Figure 17a,b). Only the railway curve exhibits a clear slope increase towards higher sound levels (beyond 60 dBA) in both annoyance outcomes. Differences in dependence of total annoyance on single source Lden vanish completely up to 60 dBA for the moderate annoyance response. Only the main road slope shows a significant increase up to 60 dBA with the highly annoyed. This increase is also significantly different at 55 (*p* = 0.0079; *p* = 0.0092) and 60 dBA (*p* = 0.018; *p* = 0.0078) from the highway and railway response. Overall, the models have a high power of explanation (pseudo R^2^ = 0.55).

**Figure 18:**
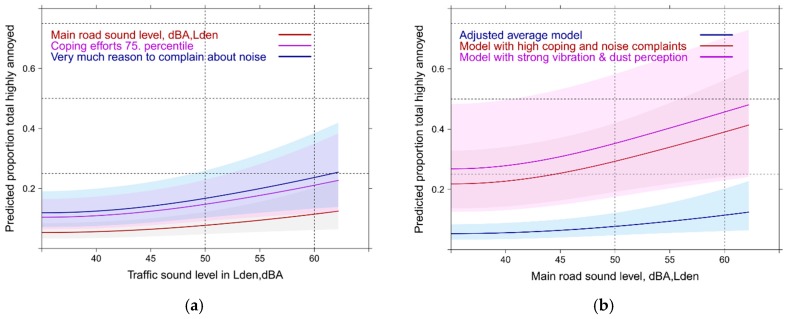
Predicted proportion of total annoyance associated with sound from all sources. (**a**) Proportion highly annoyed; (**b**) Proportion highly annoyed associated with main road. Models are adjusted for age, gender, education, health status, complaints about community soundscape and air pollution, perceived vibration, perceived dust/soot from road, coping, anger at traffic load and the respective other sound sources.

The true meaning of the contextual variables comes to the surface when the average model results are contrasted with the results of higher values on the most important model predictors (Figure 18a,b). In Figure 18a the meaning of single contextual predictors are contrasted with the meaning of the main road sound indicator in the full model including the other sound sources. Figure 18b shows the contrast with a simulation of multiple predictor combinations. For railway sound exposure the response slope increases in a similar way—but only beyond 60 dBA,Lden (not shown).

#### 3.7.2. Mixed Source Total Exposure Response Models Including Contextual Variables

**Figure 19:**
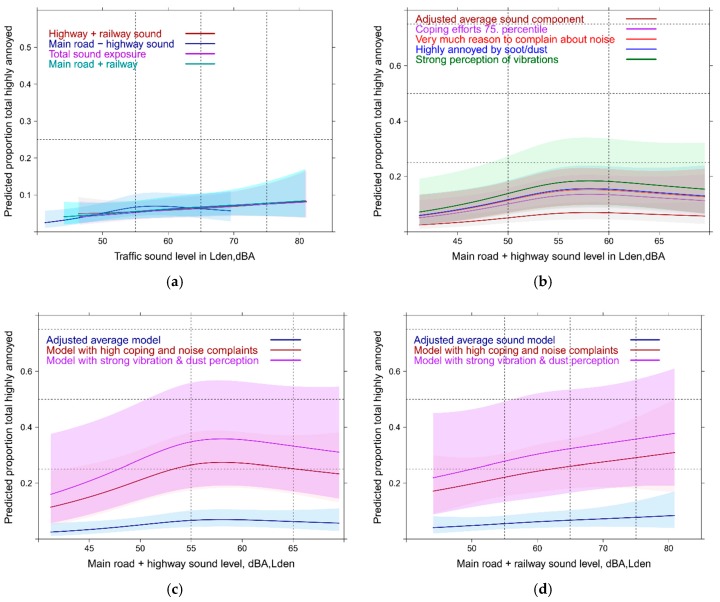
Predicted proportion of total annoyance associated with sound from mixed sources. (**a**) Proportion highly annoyed associated with the acoustical indicator; (**b**) The relative importance of predictors for the proportion highly annoyed associated with a road exposure combination; (**c**) The relative importance of predictor combinations for the proportion highly annoyed associated with a road exposure combination; (**d**) The relative importance of predictor combinations for the proportion highly annoyed associated with a main road-railway exposure combination. Models are adjusted for age, gender, education, health status. Depending on the predictors shown, adjusted for all other predictors not shown in the graph (complaints about community soundscape and air pollution, perceived vibration, perceived dust/soot from road, coping, anger at traffic load).

Similar to the full models (in Section 3.6 and Section 3.7) the dependence of total annoyance on single source Lden is weak again and difficult to distinguish (Figure 19a). A sign of the reduced importance of the acoustic variable. A slope increase is only statistically significant (*p* = 0.03) for the road combination (between 50 to 60 Lden) and borderline significant for the main road-rail combination (between 55 and 65 Lden). The pseudo R² of all models is high (0.54–0.55). 

The relative importance of the non-acoustic, and perceptional variables (Figure 19b) is evident by the stronger relation to annoyance compared to the acoustic indicator. Through a simulation of occurring predictor combinations the major impact of these predictors is underlined (Figure 19c,d).

#### 3.7.3. Full Exposure Response Models with Contextual Variables: Using the Emergence Indicators

**Figure 20:**
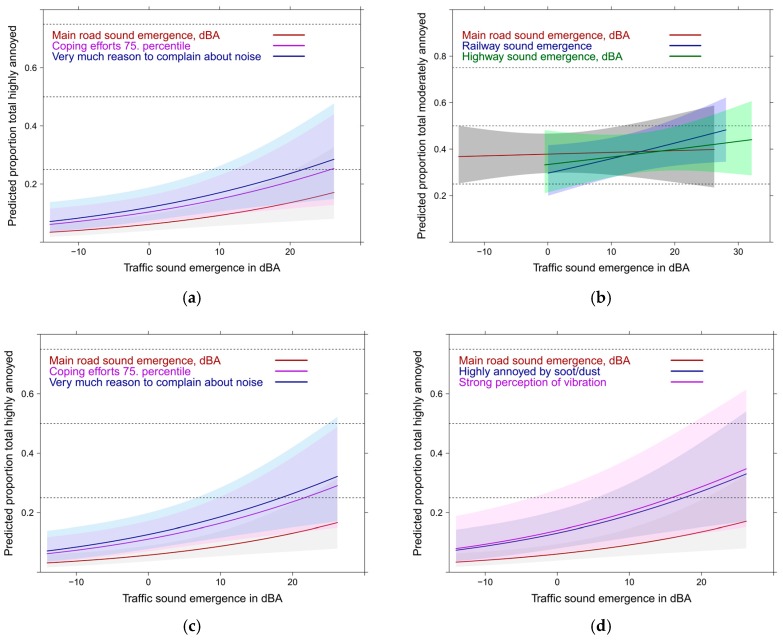
Predicted proportion of total annoyance associated with emergent sound from main roads. (**a**) Proportion highly annoyed associated with the acoustic source indicators; (**b**) Proportion moderately annoyed associated with the acoustic source indicators; (**c**) The relative importance of single behavioral predictors; and (**d**) perceptual predictors for the proportion highly annoyed associated with increasing main road emergence. Models are generally adjusted for age, gender, education, health status and emergence from other sources. Depending on the predictors shown, adjusted for other predictors not shown in the graph (complaints about community soundscape and air pollution, perceived vibration, perceived dust/soot from road, coping, anger at traffic load).

Using the full model with adjustment for all source specific adjustment only the emergence indicator withstood the collinearity assessments. In Figure 20a,b the basic relation is shown for the high and moderate annoyance outcome. Emergence of main road (*p* = 0.023) and highway (*p* = 0.023), not railway (*p* = 0.0967) show significant associations with high total annoyance. With moderate annoyance only rail emergence approaches significance (*p* = 0.056). The model pseudo R² is high in both models (0.55 and 0.58). Figure 20c,d indicates the relative importance of emergent main road sound to main predictors.

**Figure 21:**
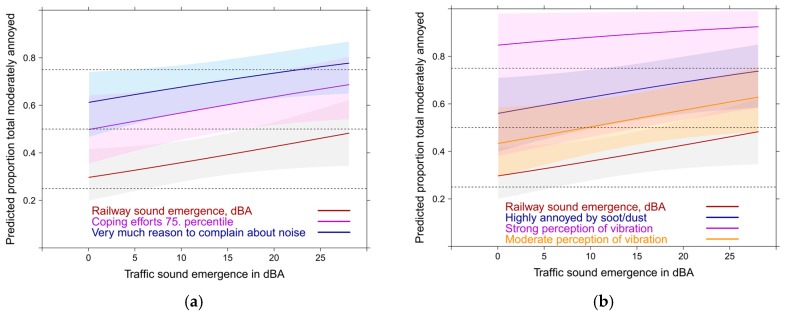
Predicted proportion of total moderate annoyance associated with the emergent sound from railways. (**a**) Proportion highly annoyed associated with the acoustic source indicators; (**b**) Proportion moderately annoyed associated with the acoustic source indicators; Depending on the predictors shown, adjusted for other predictors not shown in the graph (complaints about community soundscape and air pollution, perceived vibration (not (**b**), perceived dust/soot from road (not (**b**), coping not (**a**), anger at traffic load).

In Figure 21a,b the association of railway emergence with moderate annoyance is depicted. Notably, strong perception of vibration is associated nearly independent of the sound indicator with moderate annoyance. However, due to the uncertainty involved in the strong perception curve (small group: 4%) the relationship with moderate perception of vibration (22%) is further added to Figure 21b.

## 4. Discussion

The aim of this broader approach was twofold: first, we applied a stepwise approach with increasingly complex models and varying total annoyance endpoints (HA, MA, full scale annoyance) to evaluate the soundness of major scientific hypotheses regarding the potential impact of exposure to multiple sound sources for this sample. Secondly, in this analysis process we entered more perception related (vibration, air pollution) and other so-called non-acoustic factors to explore in addition the relative contribution of these factors in the context of a multi-source exposure situation. 

While we observed distinct Lden annoyance relationships for single source annoyance and a strong dependence of the total annoyance as a function of the single source Lden for all investigated annoyance endpoints in the unadjusted models—this changed substantially with more adjustments and the inclusion of other sound sources in the model. To the contrary, the mixed source analyses did not improve the quality of fit of the models nor indicate a consistent difference among the studied combinations regarding the relation to the total annoyance response. The exception was a significant departure of the highway-main road combination with the HA and MA outcome (Figure 2a,b) and a significant deviation by the highway-rail combination in the full scale mean annoyance analysis (Figure 5b). Notably, the total sound exposure model exhibited a much lower ER-curve and had a lower goodness of fit than the model with individual source Lden. Thus, the “combined noise source paradox” is strongly confirmed and also the energetic summation of sound sources of different character is not appropriate to assess the overall noise burden. Indirect support for this conclusion is provided also through the sensitivity of the emergence indicator in the full models.

A second observation: with increasing adjustments the dependence of total annoyance on sound level indicators gets shallower (even stronger with the MA-response). A further slope increase is seen only at the highest levels of railway exposure (Figure 17b) and/or through the additional consideration of major non-acoustic predictors (Figure 19 and Figure 20c,d).

This indicates—among other methodologic factors mentioned—limitations associated with the restrictions imposed by the energy equivalent summation of the sounds in the mixed source models and the use of the dBA-rating. e.g., the importance of low frequency (LF) components of traffic [40,41,82] could thereby not appropriately be accounted for in this analysis with classical sound indicators. The high truck traffic load of the area suggests the importance of LF assessment. Large parts of this LF-component may have been caught by the question on vibration perception [83,84,85].

Consistent in all single and multiple source models, the major contribution from non-acoustic factors was provided by the sum of coping activities (behavioral), the amount of reasons to complain about the overall community soundscape, and anger about the overall traffic load (emotional). 

On the other hand, the importance of the major perceptual factors (air pollution, vibration) associated with the sound sources varied in the models depending on source. The statistical contribution for dust/soot perception was largest with highway and main road (railway n.s.) while for vibration perception the ranking was: railway followed by main road (highway n.s.). This differentiation (Table 3) underlines specifically the validity of the questionnaire responses and the need to account for total traffic perception in future annoyance models, in planning and noise action plans.

A note on railway sound exposure: installation of noise barriers along the railway (2000–2003) reduced both sound level and also annoyance compared with the 1998 survey [86]. Thus, it seems, the rail-bonus has been retained by the abatement measures when you judge it from Figure 1a,b. This is only relative to the highest road related annoyance curves in the new WHO-data base [87]. The current railway curve shows still a higher annoyance response—compared with the EU-standard curve [88]. Thus, a significant number of houses are still exposed to high levels (5% of our sample live within 67 m, 10% within 130 m) where vibration may still be perceived. Although distance was no longer significant (*p* = 0.12) in the full contextual models, when perception of vibration was included as predictor. Thus, a close distance to railway tracks may be a factor, as Asian studies have shown [89,90], but the annoyance curves of these studies did not account for vibration perception in an model.

A potential cumulative effect of air pollution and noise on the annoyance response in near neighbors of main roads and highways is also supported by the results of Table 3. Such an effect was observed in other studies [91,92], but rarely with multiple adjustments [93] and not in a multi-source analysis.

In the sound source difference models (Figure 8, Figure 9 and Figure 10) none of the studied source difference combinations reached significance and there was no consistent pattern to be interpreted with certainty. While in one case (railway by main road) a large difference between the exposures makes a slight impact (*p* = 0.15) in other combinations it was equal or higher sound of the other source (n.s.). In the full models these difference indicator variables did not longer contribute. We believe the power to detect significant differences was even in this medium large sample too low and chance is probably also involved in the few studies which found stronger annoyance at equal sound level of both sources in a rail-highway situation [16,18,52,94,95,96]. We could find, however, in the base model with participants living in a rail-highway combination (Figure 5b) a significant higher mean annoyance response beyond 55 dBA compared with other two-source combinations. This finding would fit with one of the earlier studies. The finding could not be repeated in the parallel analysis with HA/MA as outcome. In this case, the highway-main road annoyance response exhibited a similar behavior (Figure 6a,b). Anyways, the common ground for the annoyance effects is, that most combinations are associated with a similar response and the total exposure (three sources) is not distinguishable from the other source combinations. Whether poor masking plays a significant role in multi-source sound environments such as the alpine areas with lower background levels than in urban areas remains to be proved. 

The obtained results do not imply a straight interpretation. Concerning the first objective, we have one clear-cut result: the combination of summation of sound sources with different characteristics and asking participants a (difficult) question about total annoyance by all sources results in an underestimation of the potential public health impact. There is no real support for one of the prevailing theoretical models (dominance or difference model) from the analysis of this study sample. This may be related to the fact that aircraft exposure is not an issue in this sample and we had three sources under study which have a quite distinct acoustic characteristic. Moreover, the topographic and meteorological context of an alpine valley (sound propagation over large distance versus low background sound at the slopes) is a unique feature. Thus, a direct comparison with earlier studies is difficult and a generalization should be avoided. 

The use of an emergence indicator revealed mixed results. While the indicator was a complete replacement for the classical indicator Lden, the stronger correlations with each other (compared with the Lden’s) may have introduced some residual variance inflation in the multi-source models. In the base model the results with the emergence indicator (Figure 7a,b) underlined the stressful microstructure of the main road and makes the higher annoyance response more comprehensible. On the other hand, you would have expected a stronger appearance of the likewise intermittent railway events. However, we need to inform at this point that many railway noise barriers were erected in this area as the result of an environmental health impact assessment between 1998 and 2001. This is the reason, why the annoyance response to railway is lower compared with the 1998 survey, but still higher than in the standard curves and in the updated WHO-evidence curves [87].

What can be said about the role of the four annoyance outcome measures in the analyses? The night noise outcome question obviously underestimates the real effects on sleep through its focus on behavioral awakenings. That underestimation by the night annoyance questions is a very likely reason is supported by our finding of a higher risk for sleep medications in the same data set of the ALPNAP-study [97]. Most awakenings occur unconscious without notice by the person—especially, when the exposure duration is longer than one year (which is the requirement to be included in the study).

The MA-analyses in multi-source exposure situations indicate that much more persons are annoyed already at lower levels between 40 and 50 dBA. Similar, the linear model, using the full scale (mean annoyance response), complements the findings from the MA-analyses. Overall, the public impact seem to be underestimated—specifically for the multi-source exposure situations by sole reliance upon the highly annoyed (even with the 60% cutoff used according to the ICBEN recommendation). There is also the question, whether the single annoyance question, as used in the ICBEN/ISO recommendation is enough to catch the full experience of people exposed to multiple sources. Since the international introduction of this single standard question surveys contain no longer questions on interference, which would cover a broader view of the real experienced annoyance in daily life. Proposals have been made and named it the overall affectedness [98]. 

Concerning our second objective to understand the higher annoyance response of this area we found strong support for the further importance of perceptional, emotional and behavioral variables. Moreover, the satisfaction with the wider community area in terms of air pollution and noise turned out to be of critical importance. We would not advise to interpret this information as purely related to personal factors, which are not amenable to prevention or abatement. Rather, this should be interpreted as integrated judgment related to the overall traffic soundscape [30] which indicates environmental factors which are not sufficiently accounted for by current exposure assessments. Others have outlined the implications of a wider area extended assessment of noise exposure and shown, that neglecting this extended area exposure will underestimate the overall sound load up to 15 dBA in some areas [47,99,100]. Recent studies have supported this claim and requested to expand the noise mapping strategies [100] for full exposure assessment.

The perception of dust/soot and vibration is known to enhance the annoyance response in field studies [1,92,101,102,103,104,105]. The current analysis lends strong support to earlier results [62,88], because these perceptual factors remain important in a multivariable regression after adjustment to a broad range of well-known established predictors. The correlation between the perception of dust/soot with total annoyance is higher (*r* = 0.59) than with the mean NO_2_-exposure (*r* = 0.22) or the total sound exposure (*r* = 0.14). Most other studies reported similar results from crude regression models only. Other papers have applied more complex statistical approaches to explore the wider set of influential factors [106,107,108,109,110,111]—but no exposure response data can be derived from these results.

We analyzed in detail the substantial effect of coping styles with traffic noise and odors on the annoyance response [73], however, these results are difficult to interpret in a classical exposure response framework. Now, we have additional assurance with multiple adjustments that the amount of coping efforts needed to adapt to the stress load from several sources is a critical factor to be considered in a multi-source exposure environment. Another study observed negative effects of unsuccessful coping—e.g., not closing windows—on blood pressure [39] which indicates the need to consider complex causal paths to health effects via the annoyance perspective.

The analyses in a multivariable model context confirmed also the strong deviating annoyance response towards the main road exposure reported earlier [112]. Moreover, one mixed source combination (with basic adjustments) showed a higher response—where main road was involved (Figure 6a,b). The fact, that main road neighbors rated both vibration and air pollution experience as strong, indicates a larger perceptional load compared with high- and railway: air pollution is the only significant perception factor around highways while vibration is only significant for railway areas. Eventually, the noticeability of the nearby experience of main road pass-bys in mostly rural areas [113,114], the mentioned “amphitheatre” effect towards the slope of the valley [56,57,58,115,116,117], and the visibility of the source [117,118] are possible factors responsible for the strong annoyance response. A reasonable hypothesis seems therefore that poor masking in such environments (against low background levels) contributes to the higher annoyance response. This helps to understand why the annoyance response in this alpine area is extraordinarily high for people adjacent to main roads and exceeds not only the “standard response” [88] used to assess the noise burden across the European Union and to implement action plans but represents also the highest road related annoyance curve in the WHO evidence data base [87].

Notably, we could not observe interactions (on a multiplicative scale) neither between sound sources nor between the contextual factors. One should, however, not overlook the substantial additive effects the various contextual and non-acoustic factors exert on annoyance (Figure 19, Figure 20 and Figure 21).

Overall, the study has several strengths. First, the noise exposure assessment was enriched by detailed traffic counting also from smaller nearby roads and adaptation of the sound modeling to the topographic and meteorological features. Moreover, the sound modeling was supported by week-long sound measurements under various meteorological conditions (spring, fall) with parallel recordings of meteorological parameters. We tested also alternative sound indicators (emergence, fluctuation) in a few models.

Second, the stratified, random sampling strategy (by distance to the major sources) took care to get a distribution of sufficiently large number of participants across a broad noise range for all sources. Annoyance was assessed according to the standard in the field.

Third, several annoyance outcomes were investigated. A broad range of factors known to affect the annoyance response was considered and provide hints to understand better the low “community tolerance level” (CTL) found in studies of alpine areas [31].

The cross-sectional design of the study remains the weakest point in terms of classical criteria for the interpretation of causality. Therefore, we cannot distinguish by this approach, whether annoyance causes coping and/or unsuccessful coping may result in more annoyance. This would raise the question, whether coping activities (independent of its successfulness) should be included as predictor in such a model. 

The relative small crude participation proportion (35% at individual, 50% at household level) is an underestimation due to many business addresses or secondary households in the address material. The representativity of the sample was not impaired—except for a slight excess of female participants (61%) due to their better availability on phone during call-times. The stratified sampling approach and the higher participation at the household level should also have counteracted potential distortions of the obtained exposure response results.

A few models could not be evaluated due to multi-collinearity. It cannot be excluded that the estimates for selected predictors, we adjusted for, are still affected by residual collinearity. According to the statistical literature, the outcome assessments should not be affected, except the model specification is erroneous [119]. However, the effect size of the individual predictors cannot be easily transferred to other situations with a different collinearity structure [120].

Eventually, we have not evaluated the effect of quiet sides (too small samples in multi-source environments), and visual (greenery) or other restorative factors which have shown certain annoyance reducing effects [48,50,117,118,121,122] in some subareas. However, the area variable (urban, suburban, and rural) was not a significant predictor in the full contextual model.

## 5. Conclusions

Neither the energy summation, the difference or the dominant source perspective provide an appropriate framework to address the effects of a mixed source exposure situation with three ground based traffic sources, where aircraft noise does not play a role.

Full annoyance models are needed which integrate—apart from standard adjustments for socio-demographic factors—also behavioral, perceptional, emotional, and further contextual characteristics like the community soundscape.

In multi-source situations the impact of traffic on annoyance is often underestimated for the lower exposure range (40 to 50 dBA) with the high annoyance approach. We need broader annoyance assessment questions which better catch the true overall affectedness of exposed people [98]. This includes specific questions on interference with daily activities. Those questions disappeared from survey questionnaires with the introduction of one standardized annoyance question. A reintroduction is needed to better catch the total annoyance due to multiple sources.

Moreover, acoustic indicators are needed which better characterize the specific annoyance triggering sound components [31,42,123,124]. Indicators based on average sound intensity are too highly correlated and when summed up for three sources the specific acoustic characteristic of the sources get lost. Mapping of non-A-weighted, temporally detailed and psychoacoustic indicators for larger areas is suggested as potential alternative [125]. 

Future studies, planning and environmental health impact assessments need to adopt an integrated approach [126,127] with a multisensory perspective to account for the substantial perceptual impact by vibration, air pollution, and notice events [128]. These requirements need not only to be implemented in cities, but also in rural and sensitive alpine areas to protect quiet sites, green areas for restoration and fulfill the WHO goals of the European region for supporting environments [129].

## Figures and Tables

**Table 1:** Description of categorical variables in the various models broken down by total highly annoyed.

Categorical	Total Noise Annoyance *		Sample Size	Test Statistic	
Variables	Low, *n* (%)	High, *n* (%)	Total, *n* (%)	df—Chi-Square	*p*-Value
**Full sample**	1264 (77.0)	377 (23.0)	1641 (100.0)		
**Age (years)**				Chisq. (4 df) = 17.24	0.002
25–34	211 (16.7)	45 (11.9)	256 (15.6)		
35–44	341 (27)	123 (32.6)	464 (28.3)		
45–54	303 (24)	88 (23.3)	391 (23.8)		
55–64	208 (16.5)	81 (21.5)	289 (17.6)		
65+	201 (15.9)	40 (10.6)	241 (14.7)		
**Gender**				Chisq. (1 df) = 0.03	0.86
Male	488 (38.6)	143 (37.9)	631 (38.5)		
Female	776 (61.4)	234 (62.1)	1010 (61.5)		
**Education**				Chisq. (3 df) = 9.83	0.02
Basic	211 (16.7)	53 (14.1)	264 (16.1)		
Skilled	337 (26.7)	84 (22.4)	421 (25.7)		
Vocational	310 (24.6)	121 (32.3)	431 (26.4)		
Higher	402 (31.9)	117 (31.2)	519 (31.7)		
**Health status**				Chisq. (2 df) = 24.26	<0.001
Excellent	386 (30.5)	74 (19.6)	460 (28)		
Good	449 (35.5)	129 (34.2)	578 (35.2)		
Poor	429 (33.9)	174 (46.2)	603 (36.7)		
**Noise sensitivity**				Chisq. (1 df) = 48.14	<0.001
High	158 (12.5)	104 (27.6)	262 (16)		
Low	1106 (87.5)	273 (72.4)	1379 (84)		
**Air pollution sensitivity**				Chisq. (1 df) = 89.14	<0.001
High	230 (18.2)	158 (41.9)	388 (23.6)		
Low	1034 (81.8)	219 (58.1)	1253 (76.4)		
**Annoyance by dust/soot**				Chisq. (1 df) = 340.57	<0.001
Highly annoyed	139 (11)	209 (55.4)	348 (21.2)		
Less annoyed	1125 (89)	168 (44.6)	1293 (78.8)		
**Annoyance by traffic exhaust**				Chisq. (1 df) = 247.12	<0.001
Highly annoyed	55 (4.4)	126 (33.4)	181 (11)		
Less annoyed	1209 (95.6)	251 (66.6)	1460 (89)		
**Annoyance by vibration: roads**				Chisq. (1 df) = 87.61	<0.001
Annoyed	171 (13.5)	132 (35)	303 (18.5)		
Not annoyed	1093 (86.5)	245 (65)	1338 (81.5)		
**Annoyance by vibration: railway**				Chisq. (1 df) = 33.89	<0.001
Annoyed	119 (9.4)	78 (20.7)	197 (12)		
Not annoyed	1145 (90.6)	299 (79.3)	1444 (88)		
**Area complaints: noise pollution**				Chisq. (2 df) = 300.03	<0.001
Less or no reasons to complain	426 (33.7)	23 (6.1)	449 (27.4)		
Quite a lot of reason to complain	422 (33.4)	40 (10.6)	462 (28.2)		
Very much reason to complain	416 (32.9)	314 (83.3)	730 (44.5)		
**Area complaints: air pollution**				Chisq. (2 df) = 205.83	<0.001
Less or no reasons to complain	431 (34.1)	30 (8)	461 (28.1)		
Quite a lot of reason to complain	389 (30.8)	59 (15.6)	448 (27.3)		
Very much reason to complain	444 (35.1)	288 (76.4)	732 (44.6)		
**Anger towards traffic load**				Chisq. (2 df) = 430.49	<0.001
Never	502 (39.7)	17 (4.5)	519 (31.6)		
Sometimes	527 (41.7)	81 (21.5)	608 (37.1)		
Mostly	235 (18.6)	279 (74)	514 (31.3)		
**Helpless towards traffic load**				Chisq. (2 df) = 201.41	<0.001
Never	580 (45.9)	52 (13.8)	632 (38.5)		
Sometimes	364 (28.8)	88 (23.3)	452 (27.5)		
Mostly	320 (25.3)	237 (62.9)	557 (33.9)		
**Housing: type**				Chisq. (2 df) = 5.88	0.053
appartment home	280 (22.2)	64 (17)	344 (21)		
row house	274 (21.7)	97 (25.7)	371 (22.6)		
single detached home	710 (56.2)	216 (57.3)	926 (56.4)		
**Geographic area features**				Chisq. (2 df) = 11.01	0.004
rural	340 (26.9)	90 (23.9)	430 (26.2)		
suburban	445 (35.2)	168 (44.6)	613 (37.4)		
urban	479 (37.9)	119 (31.6)	598 (36.4)		
**Traffic exposure situation: home**				Chisq. (4 df) = 50.02	<0.001
Highway within 200 m	112 (8.9)	58 (15.4)	170 (10.4)		
Railway within 200 m	148 (11.7)	74 (19.6)	222 (13.5)		
Main road within 100 m	163 (12.9)	58 (15.4)	221 (13.5)		
Mixed traffic	31 (2.5)	18 (4.8)	49 (3)		
Outside above areas	810 (64.1)	169 (44.8)	979 (59.7)		
**Annoyance by highway**				Chisq. (1 df) = 485.3	<0.001
low	1137 (90)	135 (35.8)	1272 (77.5)		
high	127 (10)	242 (64.2)	369 (22.5)		
**Annoyance by local road**				Chisq. (1 df) = 230.56	<0.001
high	1168 (92.4)	228 (60.5)	1396 (85.1)		
low	96 (7.6)	149 (39.5)	245 (14.9)		
**Annoyance by railway**				Chisq. (1 df) = 193.83	<0.001
low	1182 (93.5)	249 (66)	1431 (87.2)		
high	82 (6.5)	128 (34)	210 (12.8)		

* From all traffic sound sources; low = 1 + 2 + 3; high = 4 + 5; (verbal ISO TC 43/SC 1 2002-02 format).

**Table 2:** Statistical description of continuous variables in the various models broken down by total highly annoyed.

Continuous Variables	Total Noise Annoyance *		Sample	Test Statistic	*p*-Value
	Low (Median, IQR)	High (Median, IQR)	Total, (Median, IQR)	*t*-Test (df = 1)	
Sound level highway *				F = 44.67	<0.001
Median (IQR)	53.2 (49.1, 57.8)	56.4 (51.1,60.7)	53.7 (49.6,58.6)		
Sound level railway *				F = 45.54	<0.001
Median (IQR)	58 (52.4,62.8)	61 (54.9,66.9)	58.7 (52.9,63.9)		
Sound level main roads *				F = 16.98	<0.001
Median (IQR)	36.4 (31.3,42.2)	37.8 (33.1,46.5)	36.7 (31.7,43)		
Sound level all sources *				F = 64.63	<0.001
Median (IQR)	59.9 (54.9,64.5)	63 (58.4,68.7)	60.6 (55.5,65.7)		
**Duration of living at home: yrs**				F = 1.95	0.163
Median (IQR)	16 (7,30)	17 (8,32)	16 (7,31)		
**Annual NO_2_ level, µg/m³**				F = 31.93	<0.001
Median (IQR)	27.8 (24.8,31.3)	29.3 (26.1,34.2)	28.1 (25.1,32.1)		
**Distance to highway: m**				F = 50.75	<0.001
Median (IQR)	687.8 (385.1,1020.9)	487.3 (261.6,808.2)	631.6 (346.7,974.9)		
**Distance to main road: m**				F = 3.26	0.071
Median (IQR)	539.1 (182.5,947.4)	660.6 (191.2,1143.5)	560.6 (182.5,967.6)		
**Distance to railway: m**				F = 42.41	<0.001
Median (IQR)	681.9 (405.5,1033.6)	521 (259,787)	638 (372.5,974.9)		
**Life satisfaction score+**				F = 36.17	<0.001
Median (IQR)	30 (26,32)	28 (24,31)	29 (26,32)		
**Sleep disturbance score #**				F = 79.4	<0.001
Median (IQR)	7 (5,10)	9 (7,13)	8 (5,11)		
**Coping efforts score $**				F = 700.9	<0.001
Median (IQR)	22 (17,29)	40 (32,47)	25 (18,35)		

* From all traffic sound sources; low = 1 + 2 + 3; high = 4 + 5; (verbal ISO TC 43/SC 1 2002-02 format).

**Table 3:** The importance of perception of vibration and air pollution in traffic assessments. Extract from the logistic regression model *.

Source Model	Wald Chi-Square	df	*p*-Value
Highway-model *			
Dust/soot high	12.93	1	0.0003
Vibration high	0.63	1	0.4266
Railway-model *			
Dust/soot high	0.01	1	0.9299
Vibration high	11.05	1	0.0009
Main road-model *			
Dust/soot high	7.12	1	0.0076
Vibration high	18.96	1	<0.0001

* adjusted for age, sex, education, noise/air sensitivity, health status, complaints about noise/air, anger at traffic load, coping, satisfaction with life, house type, region, duration of living, distance to source.

## Supplementary Materials

The following are available online at www.mdpi.com/1660-4601/14/6/663/s1, Figure S1. (a) Single source sound level distribution and the number affected in the ALPNAP-sample. (rail.den.max=railway level; main.den.max=main road level; hw.den.max=highway level). (b) Multiple source sound level distribution and the number affected in the ALPNAP-sample. (mainrl.tot.En=main road-railway combination; hwrl.tot.En=highway-railway combination; tot.En=mainroad-highway-railway combination). Table S1. High annoyance model with all single sources, no adjustments (crude model), Table S2. High annoyance model with all single sources, five adjustments (base model), Table S3. High annoyance model with all single sources, full adjustments (contextual model), Table S4. Linear regression: Mean annoyance model with all single sources, 5 adjustments (base model), Table S5. Linear regression: Mean annoyance model with mixed sources, full adjustments (contextual model): Highway-railway combination, Table S6. Linear regression: Mean annoyance model with mixed sources, full adjustments (contextual model): Main road-railway combination, Table S7. Linear regression: Mean annoyance model with mixed sources, full adjustments (contextual model): Three source combination (main road, highway, and railway), Table S8. Logistic regression: High annoyance model with emergence indicators for all sources, full adjustments (contextual model), Supplementary: Annoyance questionnaire.
