# Supplementary material for: Community Response to Multiple Sound Sources: Integrating Acoustic and Contextual Approaches in the Analysis
Source: Int J Environ Res Public Health. 2017 Jun 20;14(6):663. doi: 10.3390/ijerph14060663 (PMC5486349; doi:10.3390/ijerph14060663)
Supplement: Supplementary file 1 [file ijerph-14-00663-s001.pdf]

International Journal of
Environmental Research and Public Health

MDPI

Community Response to Multiple Sound Sources: Integrating Acoustic and Contextual Approaches in the Analysis

Peter Lercher, Bert De Coensel, Luc Dekonink and Dick Botteldooren

Supplementary Material

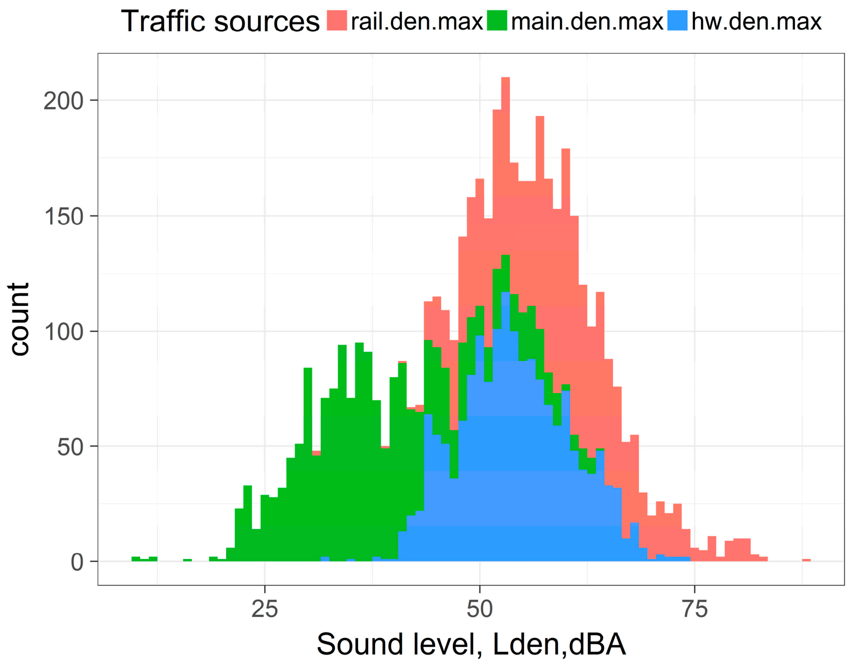

(a)

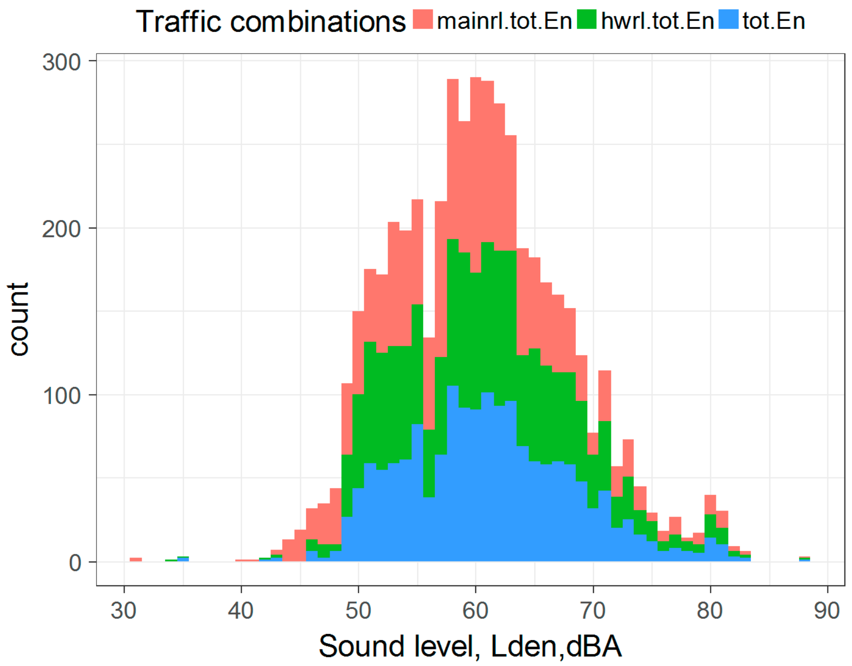

(b)

Figure S1. (a) Single source sound level distribution and the number affected in the ALPNAP-sample. (rail.den.max=railway level; main.den.max=main road level; hw.den.max=highway level). **(b)** Multiple source sound level distribution and the number affected in the ALPNAP-sample. (mainrl.tot.En=main road-railway combination; hwrl.tot.En=highway-railway combination; tot.En=mainroad-highway-railway combination).

Below are the Anova tables of selected models to illustrate the differences among the various models expressed in numbers for the interested reader.

Table S1. High annoyance model with all single sources, no adjustments (**crude model**).

Predictor	Wald chi-square	df	P-value
Main road, Lden	36.37	2	<.0001
Nonlinear	6.37	1	0.0116
Railway, Lden	16.79	2	0.0002
Nonlinear	2.91	1	0.0878
Highway, Lden	11.03	2	0.0040
Nonlinear	0.17	1	0.6824
TOTAL NONLINEAR	13.49	3	0.0037
TOTAL	91.79	6	<.0001
$R^2 = 0.085$	corresponding with Figure 1a		

Table S2. High annoyance model with all single sources, five adjustments (**base model**).

Predictor	Wald chi-square	df	P-value
Main road, Lden	31.03	2	<.0001
Nonlinear	5.31	1	0.0212
Railway, Lden	15.04	2	0.0005
Nonlinear	2.38	1	0.1227
Highway, Lden	12.90	2	0.0016
Nonlinear	0.45	1	0.5025
Age (yrs)	0.79	1	0.3728
Sex	0.06	1	0.8037
Education (3 levels)	9.65	2	0.0080
Noise sensitivity (2 levels	35.65	1	<.0001
Health status (3 levels)	19.06	2	0.0001
TOTAL NONLINEAR	12.16	3	0.0069
TOTAL	146.04	13	<.0001
$R^2 = 0.145$	corresponding with Figure 4a		

Table S3. High annoyance model with all single sources, full adjustments (**contextual model**).

**Predictor**	**Wald chi-square**	**df**	***p*-value**
Main road, Lden	7.65	2	0.0218
Nonlinear	2.50	1	0.1141
Railway, Lden	2.50	2	0.2872
Nonlinear	2.28	1	0.1307
Highway, Lden	2.02	2	0.3637
Nonlinear	1.25	1	0.2641
Age (yrs)	0.19	1	0.6642
Education (3 levels)	3.95	2	0.1386
Health status (3 levels)	2.58	2	0.2757
Complaints about soundscape (4 levels)	22.07	1	<.0001
Complaints about air pollution (4 levels)	5.00	1	0.0253
Dust/soot (high vs low perception)	25.14	1	<.0001
Anger at traffic load (4 levels)	25.00	2	<.0001
Sum of coping	42.26	1	<.0001
Vibration (3 levels of percpetion strength)	6.73	2	0.0346
TOTAL NONLINEAR	4.97	3	0.1739

TOTAL	380.98	19	<.0001
$R^2 = 0.55$	corresponding with Figure 17a		

Table S4. Linear regression: Mean annoyance model with all single sources, 5 adjustments (**base model**).

Predictor	df	Partial SS	MS	F	P-value
Main road, Lden	2	47.5698820	23.7849410	16.56	<.0001
Nonlinear	1	6.1654770	6.1654770	4.29	0.0384
Railway, Lden	2	34.2210170	17.1105090	11.91	<.0001
Nonlinear	1	2.5818480	2.5818480	1.80	0.1802
Highway, Lden	2	24.1817950	12.0908970	8.42	0.0002
Nonlinear	1	5.7555950	5.7555950	4.01	0.0455
Age (yrs)	1	4.2364300	4.2364300	2.95	0.0861
Sex	1	3.3540760	3.3540760	2.34	0.1267
Education (3 levels)	2	20.2811820	10.1405910	7.06	0.0009
Noise sensitivity (2 levels)	1	51.5983800	51.5983800	35.93	<.0001
Health status (3 levels)	2	47.8697220	23.9348610	16.67	<.0001
TOTAL NONLINEAR	3	25.7604320	8.5868110	5.98	0.0005
REGRESSION	13	341.3753750	26.2596440	18.28	<.0001
ERROR	1627	2336.7087200	1.4362070		
$R^2= 0.127$	Figure not shown in the paper				

Table S5. Linear regression: Mean annoyance model with mixed sources, full adjustments (**contextual model**): Highway-railway combination.

Predictor	df	Partial SS	MS	F	P-value
Highway-railway combination, Lden	2	3.89057738	1.94528869	2.84	0.0589
Nonlinear	1	0.07748352	0.07748352	0.11	0.7368
Age (yrs)	1	1.20767088	1.20767088	1.76	0.1846
Education (3 levels)	3	2.10322796	0.70107599	1.02	0.3816
Current health status (3 levels)	1	0.43386264	0.43386264	0.63	0.4264
Complaints about soundscape (4 levels)	2	3.46531096	1.73265548	2.53	0.0802
Complaints about air pollution (4 levels)	1	84.28729251	84.28729251	122.95	<.0001
Dust/soot (high vs low perception)	1	2.30552534	2.30552534	3.36	0.0669
Anger at traffic load (4 levels)	1	32.95392214	32.95392214	48.07	<.0001
Sum of coping	2	42.95513104	21.47756552	31.33	<.0001
Vibration (3 levels of percpetion strength)	1	72.38118332	72.38118332	105.58	<.0001
vibra.f3	2	15.90078692	7.95039346	11.60	<.0001
REGRESSION	17	1565.42789904	92.08399406	134.32	<.0001
ERROR	1623	1112.65619602	0.68555527		
$R^2=0.585$	corresponding with Figure 15a				

Table S6. Linear regression: Mean annoyance model with mixed sources, full adjustments (**contextual model**): Main road-railway combination.

Predictor	df	Partial SS	MS	F	P-value
Main road-railway combi	2	5.8118749	2.9059375	4.25	0.0145
Nonlinear	1	0.4317622	0.4317622	0.63	0.4271
Age (yrs)	1	1.1421510	1.1421510	1.67	0.1966
Education (3 levels)	3	2.1001728	0.7000576	1.02	0.3814
Noise sensitivity (2 levels	1	0.4362432	0.4362432	0.64	0.4248
Health status (3 levels)	2	3.5949591	1.7974795	2.63	0.0726
Complaints about soundscape (4 levels)	1	82.7782681	82.7782681	120.96	<.0001
Complaints about air pollution (4 levels)	1	2.6338978	2.6338978	3.85	0.0500
Dust/soot (high vs low perception)	1	32.6811489	32.6811489	47.75	<.0001
Anger at traffic load (4 levels)	2	43.1152602	21.5576301	31.50	<.0001
Sum of coping	1	71.9844539	71.9844539	105.18	<.0001
Vibration (3 levels of percpetion)	2	14.7862237	7.3931118	10.80	<.0001

REGRESSION	17	1567.3491966	92.1970116	134.72	<.0001
ERROR	1623	1110.7348985	0.6843715		
R^2=0.585		corresponding with Figure 15a			

Table S7. Linear regression: Mean annoyance model with mixed sources, full adjustments (**contextual model**): Three source combination (main road, highway, and railway).

Predictor	df	Partial SS	MS	F	P-value
Three source combination	2	4.6398719	2.3199360	3.39	0.0341
Nonlinear	1	0.2187568	0.2187568	0.32	0.5721
Age (yrs)	1	1.2082340	1.2082340	1.76	0.1844
Education (3 levels)	3	2.1158149	0.7052716	1.03	0.3785
Noise sensitivity (2 levels	1	0.4164399	0.4164399	0.61	0.4357
Health status (3 levels)	2	3.4949605	1.7474802	2.55	0.0783
Complaints about soundscape (4 levels)	1	82.4294748	82.4294748	120.32	<.0001
Complaints about air pollution (4 levels)	1	2.4549832	2.4549832	3.58	0.0585
Dust/soot (high vs low perception)	1	32.6868603	32.6868603	47.71	<.0001
Anger at traffic load (4 levels)	2	43.1575022	21.5787511	31.50	<.0001
Sum of coping	1	71.9847253	71.9847253	105.07	<.0001
Vibration (3 levels of percpetion)	2	15.4682053	7.7341027	11.29	<.0001
REGRESSION	17	1566.1771936	92.1280702	134.48	<.0001
ERROR	1623	1111.9069015	0.6850936		
R^2=0.585		corresponding with Figure 15a			

Table S8. Logistic regression: High annoyance model with emergence indicators for all sources, full adjustments (**contextual model**).

Predictor	Wald chi-square	df	P-value
Emergence indicator highway*	5.68	1	0.0171
Emergence indicator main road*	9.28	1	0.0023
Emergence indicator railway*	2.76	1	0.0967
Age (yrs)	0.60	1	0.4372
Education (3 levels)	4.77	2	0.0922
Health status (3 levels)	2.81	2	0.2453
haustyp.3	3.35	2	0.1869
Complaints about soundscape (4 levels)	21.45	1	<.0001
Complaints about air pollution (4 levels)	4.57	1	0.0325
Dust/soot (high vs low perception)	24.45	1	<.0001
Anger at traffic load (4 levels)	25.08	2	<.0001
Sum of coping	41.88	1	<.0001
Vibration (3 levels of perception strength)	7.37	2	0.0251
TOTAL	381.69	18	<.0001
R^2 = 0.552	corresponding with Figure 20 a,c,d		

* See methods section for definition: 2.2. Sound Exposure Assessment

Annoyance questionnaire

ICBEN questionnaire on annoyance

How annoyed/disturbed were you during the past 12 months in your home and your immediate sourrounding (Garden etc.) BY ....

Not at all annoyed/disturbed	**0—1—2—3—4—5—6—7—8—9—10**	Extremely annoyed/disturbed

Item	0	1	2	3	4	5	6	7	8	9	10
38. durch den Lärm **INSGESAMT**<br>by noise overall	☐	☐	☐	☐	☐	☐	☐	☐	☐	☐	☐
39. durch die Luftverschmutzung **INSGESAMT**<br>by air pollution overall	☐	☐	☐	☐	☐	☐	☐	☐	☐	☐	☐
40. durch Lärm von der Autobahn<br>by noise from highway	☐	☐	☐	☐	☐	☐	☐	☐	☐	☐	☐
41. durch Lärm vom Lokalverkehr<br>by noise from local roads	☐	☐	☐	☐	☐	☐	☐	☐	☐	☐	☐
42. durch Lärm vom Schienenverkehr<br>by noise from railways (underground/tramways)	☐	☐	☐	☐	☐	☐	☐	☐	☐	☐	☐
43. durch Erschütterungen vom Schienenverkehr<br>by vibration from railways (underground/tramways)	☐	☐	☐	☐	☐	☐	☐	☐	☐	☐	☐
44. durch Abgase vom Straßenverkehr<br>by traffic exhaust from road traffic	☐	☐	☐	☐	☐	☐	☐	☐	☐	☐	☐
45. durch Staub, Russ vom Straßenverkehr<br>by soot/dust from road traffic	☐	☐	☐	☐	☐	☐	☐	☐	☐	☐	☐
46. durch Fluglärm<br>by noise from aircraft	☐	☐	☐	☐	☐	☐	☐	☐	☐	☐	☐
47. durch Nachbarschaftslärm<br>by noise from neighbours	☐	☐	☐	☐	☐	☐	☐	☐	☐	☐	☐
48. durch Betriebslärm<br>by noise from businesses/industrial facilities	☐	☐	☐	☐	☐	☐	☐	☐	☐	☐	☐
49. durch Baulärm<br>by noise from construction	☐	☐	☐	☐	☐	☐	☐	☐	☐	☐	☐
50. durch Lärm von Freizeiteinrichtungen (Sportplätze, etc.)<br>by noise from sports and leasure facilities	☐	☐	☐	☐	☐	☐	☐	☐	☐	☐	☐
51. durch Luftverschmutzung durch Betriebe, Industrie<br>by air pollution from businesses/industrial facilities	☐	☐	☐	☐	☐	☐	☐	☐	☐	☐	☐
52. durch Erschütterungen durch Betriebe, Industrie<br>by vibrations from businesses/industrial facilities	☐	☐	☐	☐	☐	☐	☐	☐	☐	☐	☐

Source: BBT & ALPNAP-studies, Medical University of Innsbruck

SLEEP SCALE

During the past three months did you experience the following sleep problems?

	Never	Rarely	Several times/months	Several times/week	Almost daily
Problems falling asleep	☐	☐	☐	☐	☐
Wakening up often	☐	☐	☐	☐	☐
Problems falling back to sleep	☐	☐	☐	☐	☐
Too early awakening	☐	☐	☐	☐	☐
Tiredness/fatigue in the morning	☐	☐	☐	☐	☐

Source: BBT & ALPNAP-studies, Medical University of Innsbruck

EUROBAROMETER SCALE: Noise & air pollution

Q.5. Where you live, do you have very much reason, quite a lot of reason, not very much reason or no reason at all to complain about... ? (SHOW CARD WITH SCALE)

READ OUT		Very much reason	Quite a lot of reason	Not very much reason	No reason at all	DK
1. the quality of tap water (M)	29	1	2	3	4	5
2. the quality of water for swimming (N)	30	1	2	3	4	5
3. noise	31	1	2	3	4	5
4. air pollution	32	1	2	3	4	5
5. waste disposal	33	1	2	3	4	5
6. lack of green spaces	34	1	2	3	4	5
7. damage done to the landscape	35	1	2	3	4	5
8. traffic problems (M)	36	1	2	3	4	5
9. the quality of food products (N)	37	1	2	3	4	5
10. the organisation of civil defence in the face of natural or technological disasters (floods, earthquakes, fires, etc.) (N)	38	1	2	3	4	5

Source: EUROBAROMETER 51.1 APRIL-MAY 1999, CODEBOOK, INRA (EUROPE)
